# Cyndi: a multi-objective evolution algorithm based method for bioactive molecular conformational generation

**DOI:** 10.1186/1471-2105-10-101

**Published:** 2009-03-31

**Authors:** Xiaofeng Liu, Fang Bai, Sisheng Ouyang, Xicheng Wang, Honglin Li, Hualiang Jiang

**Affiliations:** 1 Drug Discovery and Design Center, State Key Laboratory of Drug Research, Shanghai Institute of Materia Medica, Chinese Academy of Sciences, Shanghai 201203, PR China; 2 Department of Engineering Mechanics, State Key Laboratory of Structural Analysis for Industrial Equipment, Dalian University of Technology, Dalian 116023, PR China; 3 School of Pharmacy, East China University of Science and Technology, Shanghai 200237, PR China; 4 Department of Chemical Engineering, Dalian University of Technology, Dalian 116012, PR China

## Abstract

**Background:**

Conformation generation is a ubiquitous problem in molecule modelling. Many applications require sampling the broad molecular conformational space or perceiving the bioactive conformers to ensure success. Numerous *in silico *methods have been proposed in an attempt to resolve the problem, ranging from deterministic to non-deterministic and systemic to stochastic ones. In this work, we described an efficient conformation sampling method named Cyndi, which is based on multi-objective evolution algorithm.

**Results:**

The conformational perturbation is subjected to evolutionary operation on the genome encoded with dihedral torsions. Various objectives are designated to render the generated *Pareto optimal *conformers to be energy-favoured as well as evenly scattered across the conformational space. An optional objective concerning the degree of molecular extension is added to achieve geometrically extended or compact conformations which have been observed to impact the molecular bioactivity (*J Comput -Aided Mol Des *2002, **16: **105–112). Testing the performance of Cyndi against a test set consisting of 329 small molecules reveals an average minimum RMSD of 0.864 Å to corresponding bioactive conformations, indicating Cyndi is highly competitive against other conformation generation methods. Meanwhile, the high-speed performance (0.49 ± 0.18 seconds per molecule) renders Cyndi to be a practical toolkit for conformational database preparation and facilitates subsequent pharmacophore mapping or rigid docking. The copy of precompiled executable of Cyndi and the test set molecules in mol2 format are accessible in Additional file 1.

**Conclusion:**

On the basis of MOEA algorithm, we present a new, highly efficient conformation generation method, Cyndi, and report the results of validation and performance studies comparing with other four methods. The results reveal that Cyndi is capable of generating geometrically diverse conformers and outperforms other four multiple conformer generators in the case of reproducing the bioactive conformations against 329 structures. The speed advantage indicates Cyndi is a powerful alternative method for extensive conformational sampling and large-scale conformer database preparation.

## Background

One of the imperative aspects in drug design and development is to perceive corresponding bioactive conformations which determine the physical and biological properties of drugs [1]. Conformation generation is the kernel in computer-aided drug design (CADD) methods such as molecular docking [2-4], pharmacophore construction and matching [5,6], 3D database searching [7-9], 3D-QSAR [10-12], and molecular similarity/dissimilarity analysis [13], to name a few. The ability to account for conformational flexibility is highly valued by these methods as it presumes that small molecules have to adopt energy-reasonable conformations in respect of different environments. However, according to Boltzmann Law, the properties observed for "one molecule" are actually the conformer-ensemble averages [14]. The conformers with high energies contribute little to the ensemble-average properties quantitatively and consequently have to be discarded during the conformation generation process. To select those low-energy conformers, a brute-force method can be applied to enumerate a set of conformations to describe the real-life distribution of the molecular conformational ensemble across the energy surface. Unfortunately, thorough conformational sampling may lead to combinatorial explosion problem even if the molecules are decomposed into fragments first and recombined into new conformers using predefined torsion library [15]. Therefore, a practical conformational ensemble should guarantee the conformers are energy reasonable and can span available conformational space evenly.

Recent studies on crystal structures of ligand-protein complexes revealed that the bioactive molecules tend to adopt more extended conformations than compact ones [16] and may be several kcal/mol higher in energy than their respective global energy minima [17]. Although our knowledge about the pharmacologically allowed conformational space is still limited, one of the criteria for accessing conformer generation tools remains to be to what extent the experimental determined conformations can be reproduced as quickly as possible since it's not applicable to cover the whole conformational space in short time. Researchers are referred to the works by Bostrom who evaluated the capability of reproducing the bioactive conformations of several state-of-art conformation generation programs [18-20]. When it comes to conformational analysis in which multiple low-energy conformations are required, the generated conformers need to be geometrically distinct in case that some "hot spots" of the conformational space are over sampled, which cannot reflect the molecular flexibility because duplicated conformers failed to provide new information about the system. From this point of view, conformation generation may be formulated as a multi-objective optimization process in which the optima are not dominated by sole criteria exclusively. Moreover, besides of potential energy and geometrical diversity restraints, other sophisticated or rule-of-thumb criteria such as pharmacophore and binding pocket mapping can be implemented to sample more biased conformers fulfilling these objectives.

As a non-deterministic optimization method, genetic algorithm (GA) has been broadly applied in molecular docking, pharmacophore construction, and conformation generation [21-31]. Most traditional GA implementations of conformation generation perturb the dihedral torsions of rotatable bonds (sometimes plus flipping the ring conformations by modifying the geometry of sp^3 ^hybridized atoms at the junctions of two (or more) fused aliphatic rings) using single objective function, which can be either potential energy of single individual conformers or some sophisticated designed metric of conformational space completeness/saturation in conjunction with the population each individual belongs to. For a detailed review of application of single objective GA in computer-aided drug design see the references [32,33]. However, in practical application, an ideal conformer ensemble must meet both energy and diversity criteria simultaneously (even though they conflict to each other sometimes), and it's imperative to consider all the objectives to find a set of equally valid optima (namely *Pareto-optimal *solutions) to maintain the balance between these restraints. Consequently multi-objective genetic algorithm (MOGA) or multi-objective evolution algorithm (MOEA) is an alternative appropriate method to solve such trade-off problems.

MOGA or MOEA method has been successfully applied to diverse areas in CADD, e.g. molecular docking [34], pharmacophore generation [35,36], combinational library building [37], and QSAR analysis [38]. During the preparation of this manuscript, Vainio used the MOGA method to generate conformer ensembles for drug-size organic molecules [39]. In his work, two uncorrelated objective functions, van de Waals (VDW) energy and dihedral torsion energy, were calculated individually using the MMFF94 force field. A niche filter on the basis of calculating the root mean square deviation (RMSD) between conformers as well as a user-definable energy cutoff value were used to reduce the size of the generated ensemble in the post processing step. Tested against the CCDC/Astex test set containing 311 entries with known bioactive conformations, the authors declared their method successfully produced low-energy conformers that are geometrically distinct from each other, which was the result their method designed to obtain.

In this work, a MOEA-based conformation generation method, named Cyndi, is presented with four specially designed objective functions. The conformers are encoded into GA individuals with the dihedral torsions of rotatable bonds, VDW and torsional energy terms are selected as two distinctive objectives to separate the generated conformers in energy space using Tripos force field [40]; moreover, the geometric dissimilarity (GD) of the individuals is characterized by comparing the RMSD value between each individual and the corresponding initial conformer. As an optional objective, the molecular gyration radius is employed to sample the conformations with various geometric extension degrees. As a proof-of-concept test, a combined test set composed of 329 small molecules, whose bioactive conformers are available from Protein Data Bank (PDB) [41], is benchmarked against our method. The ability of Cyndi in reproducing bioactive conformations as well as generating geometrically diverse conformational ensembles is well assessed and the results demonstrate that Cyndi provides an effective means to sample the conformational space and find the set of conformers with expected energy stability and geometric diversity.

## Results and discussion

### Flowchart

**Figure 1:**
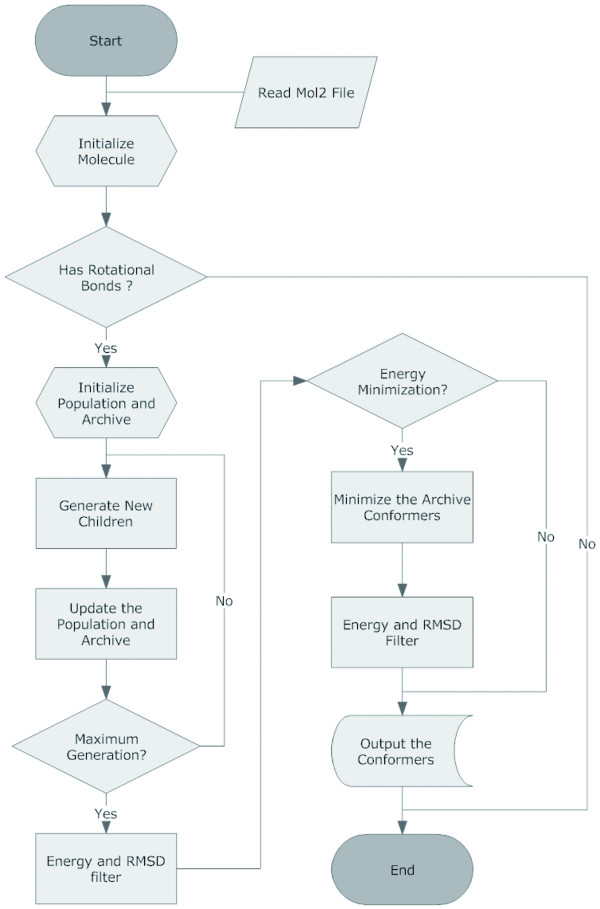
**Flowchart of Cyndi method**.

The design and application of the genomes and objectives are based on *ο*-MOEA method developed by Deb *et al*. [42]. The general flowchart of Cyndi is elucidated in Figure 1 and summarized below:

a. Read the input molecules, and then initialize the population of *N *individuals by perturbing the input 3D conformer with randomly generated dihedral angles. Afterwards, step through the population to pick up the non-dominant individuals into archives.

b. Pick up the parents using tournament selection to generate new child with crossover and mutation operations, calculate the fitness for the new child and update the archive if the new child dominates any individuals in the archive. Repeat the generation procedure until the termination condition is achieved.

c. Discard the geometrical redundant conformers with the RMSD filter.

d. Optimize the remaining conformers with energy minimization and discard the conformers outside the energy cutoff, then discard the geometrical redundant conformers again.

e. Output the final generated conformers.

### Profiles of conformer ensembles generated by MOEA method

With traditional single-objective approach, the solutions identified through several independent runs may be local minimum in a single-objective space. Hence, compromise between energy and geometrical feasibility has to be explicitly considered to select the solutions from the single-objective conformational space [37]. Cyndi surmounted this problem by offering a set of *pareto *solutions from which the users can select a conformer bearing favoured VDW energy but low GD value or vice versa. In this way the *Pareto *frontier actually covers the different region of the solution space and suppresses the "randomness" inherited with the EA algorithm. Different conformations are generated and possess rationality with respect to at least one objective.

**Figure 2:**
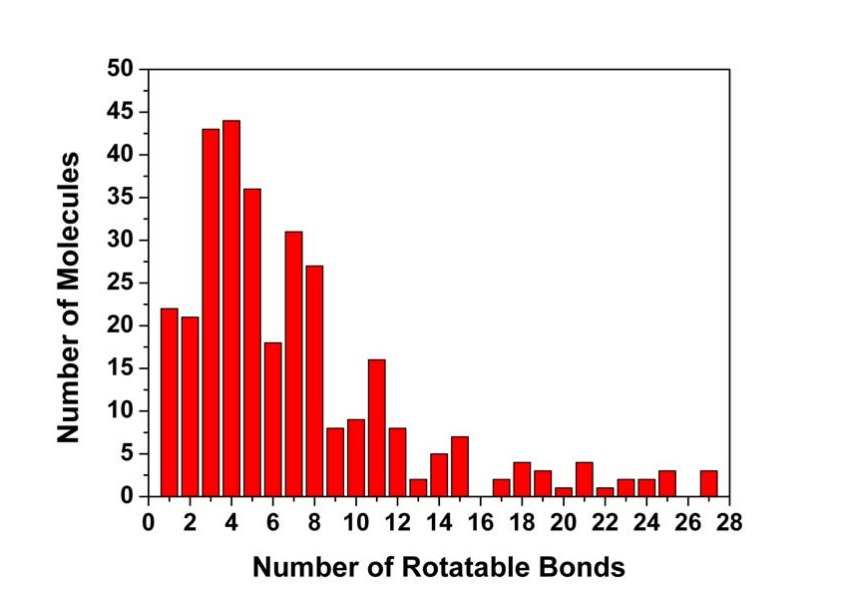
**Profile of solution space of the final archive from the ligands of PDB entry **1MCR. A total of 65 solutions are reserved in the archive. (a) 3D grid surface landscape of Pareto solutions projected on the 3-objective space. The colors are varied according to the Geometric Dissimilarity (GD, represented by RMSD to initial conformation) values as the color bar legend labelled. Three low GD valleys are labelled as the black arrows. (b) 3D scatter points representation of the Pareto solutions in 3-objective space. (c) 2D contour representation of Pareto solutions in 3-objective space. The colors are deepened as the GD values increase. The 65 conformers are projected on the space with black points, whose size varies according to their GD values respectively. The overlapped solutions are marked with red ovals as outliers.

For the case with 2-objective functions, the *Pareto optimal *solutions in the final archive should distribute across a 2D *Pareto *frontier curve and non-overlap with each other. For the case with 3-objective functions, the *Pareto *front curve transformed into a 3D surface whose profile is hard to predict. Therefore, to visualize the distribution of the solutions on the *Pareto *front, the exemplary scatter points as well as surface landscape for the ligand of 1mcr (PDB ID) is plotted in the 3D space composed of 3 objective values of each solution in the final archive (see Figures 2a and 2b). To clarify the result, the 3D hypersurface are mapped onto the plane formed by the other two objectives in contour mode that using continuous deepening colors to represent increasing GD values (see Figure 2c). Then corresponding solutions are also mapped on the same plane as scattered points whose sizes varied with corresponding RMSD values. One can see the solutions in the final archive are separated quite well in the three-objective space and only few points overlapped on the solution surface (marked with red ovals as outliers). These solutions are the representatives of the feasible solution space.

### Computational Performance of Cyndi

**Figure 3:**
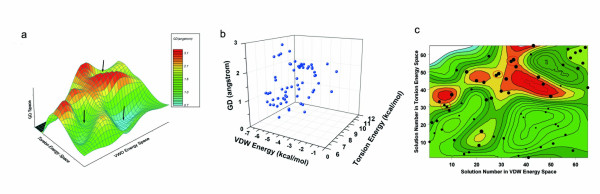
**Distribution of rotatable bonds in the test set containing 329 molecular structures**.

**Figure 4:**
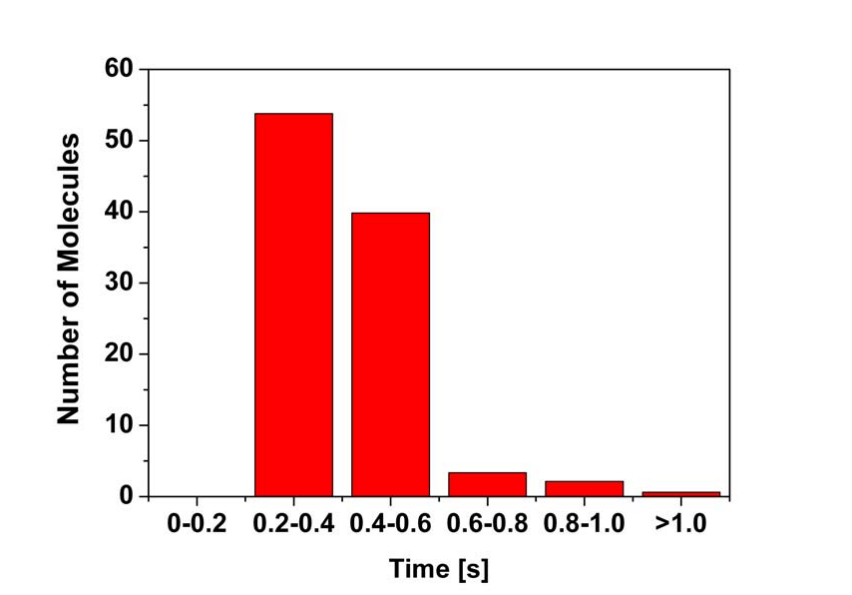
**Distribution of CPU time used in MOEA procedure for all 329 conformer ensembles**.

The ability for conformation generation of Cyndi was tested against a data set containing 329 drug-sized molecules whose bioactive conformations were extracted from the PDB database (see Methods Section). Figure 3 presents the distribution of the rotatable bonds of the test set, the PDB IDs and rotatable bonds numbers corresponding to each molecule are listed in Table A1 (see Additional file 2). Most of the molecules posses less than 11 rotatable bonds. To access the computational cost of Cyndi, the histogram of processing times is presented in Figure 4. For the overwhelming majority of the molecules, the conformation generation time distributes from 0.2 seconds to 0.6 seconds and the average computation time is about 0.49 ± 0.18 seconds per molecule. The computational cost of post minimization was not considered during the evaluation because energy minimization is the major time limit step for most stochastic conformational analysis algorithms and intensively depends on the applied local optimization algorithms, adopted force field, and specified minimization iterations. Alternatively the conformer ensembles generated with Cyndi can also be energy minimized by external softwares implemented with alternative force fields, therefore the energy optimization is discarded from computational time evaluation. As a matter of fact, the average computational time of Cyndi amounts to 23.68 seconds and never exceeds 50 seconds for each molecular conformer ensemble in an independent run, which is still acceptable and superior to that of Balloon or Catalyst Best (at least 100 seconds per ligand conformer ensemble averagely). The result presented here proves that Cyndi is extremely fast and capable of generating conformers in nearly constant time disregarding the molecular flexibility, and therefore, can be utilized to prepare conformational database for large-scale molecular database used in virtual screening.

**Figure 5:**
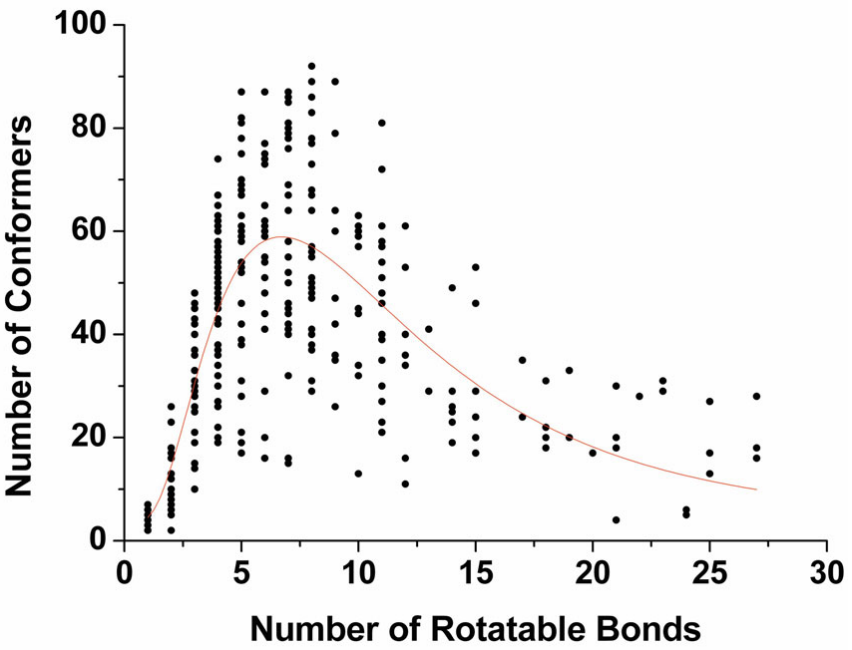
**Number of generated conformers per molecule plotted against the number of the rotatable bonds**. The observed skewed peak distribution trend is represented in the solid line.

Cyndi is originally designed as an "upstream" conformation generator for ligand-based virtual screening in the pipeline of computer-aided drug design. The numbers of generated conformers have to be limited due to the fact that "downstream" calculations are probably highly computationally expensive. Theoretically the sizes of the conformational ensembles may increase significantly for highly flexible molecules. The number of conformers obtained after post processing are plotted against the number of rotatable bonds of each molecules (Figure 5). Interestingly, the numbers of generated conformers don't increase linearly with the numbers of rotatable bonds as expected, instead, a skewed distribution with a peak value of 7 rotatable bonds was observed (as plotted in grey line). For highly flexible molecules with more than 20 rotatable bonds, the RMSD filter scaled on number of rotatable bonds tends to drive the geometric similarity criterion more stringent by increasing RMSD tolerance. Therefore the conformational space in the archive is considered to be more "crowded" and more conformers were discarded for the highly flexible molecules. This treatment was a trade-off between elimination of conformational "combinatorial explosion" and enumeration of excessive geometrically diverse conformers. Cyndi employs fixed population size, and if one hopes to sample the conformational space for the flexible molecules more thoroughly, multiple runs or increased population size is recommended. Cyndi combines the *Pareto optimal *conformers from independent runs and outputs final conformation ensemble after energy as well as geometric filter.

### Assesing the ability of reproducing bioactive conformations of Cyndi

**Figure 6:**
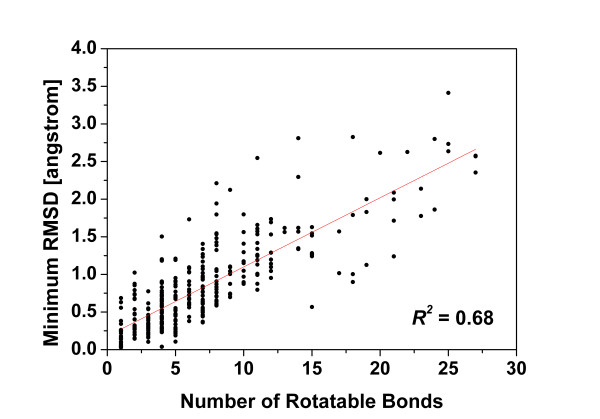
**Minimum RMSD by superposition of generated conformers and crystal bound conformation plotted against the number of rotatable bonds**. The observed linear trend is presented in solid line.

**Figure 7:**
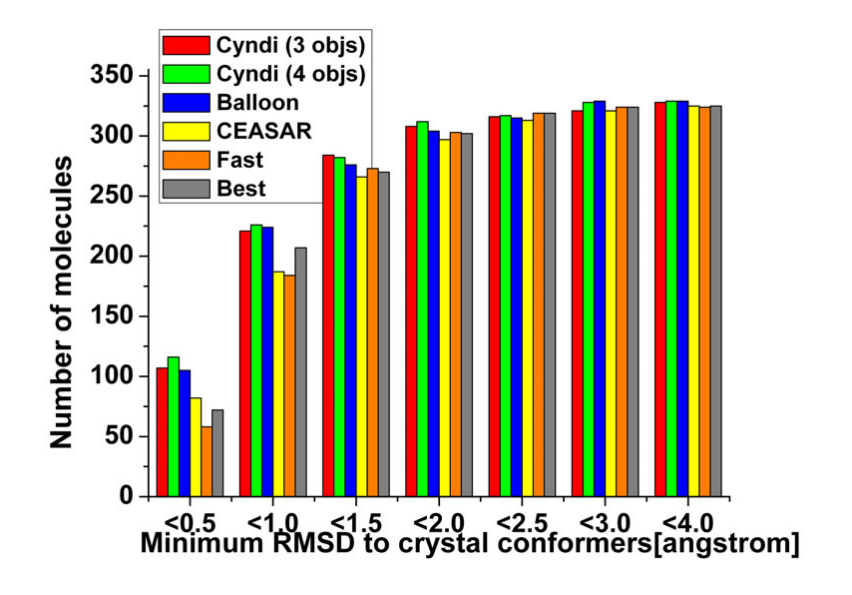
**The distribution of best-fit conformers (defined by minimum RMSD to crystal structures) to 329 compounds for Cyndi, Catalyst Fast, Catalyst Best, Catalyst CEASAR and balloon**. The best fit RMSD cumulative distributions for 329 molecules are compared among 6 methods: Cyndi (both with 3 and 4 objectives), Catalyst Fast, Catalyst Best, Catalyst CEASAR and Balloon. Note that Fast, Best and CEASAR failed to generate conformations for some molecules, as the entries labeled by "NA" in Table A1 (see Additional file 2).

To evaluate Cyndi's capability of reproducing the bioactive crystal structures, the distribution of minimum RMSD values between best-fit conformers and crystal structures is shown in Figure 6 by plotting against the number of rotatable bonds of corresponding molecules. The minimum RMSD values are linearly correlated to the numbers of rotatable bonds of ligands with *R*^2 ^= 0.68, which is in accordance to the expectation that the predictive inaccuracy increases with the molecular flexibility. The average minimum RMSD value of total 329 conformational ensembles to their crystal bound conformers is 0.864 ± 0.687 Å for 3-objective case (0.831 ± 0.601 Å for 4-objective case), and in addition, nearly 67.2% of the minimum RMSD values are below 1.0 Å (93.6% below 2.0 Å). As a comparison, both the results of Cyndi with and without the optional objective (gyration radius) are compared with Balloon, and three conformation generation methods provided in Catalyst. Figure 7 shows the histogram of the cumulative distribution of the 329 molecules whose minimum RMSD to crystal conformers is within specific cutoff thresholds. It's apparent that the results of Cyndi are better or equal to those of the 4 methods considering that they share similar percentage of conformers within 2 Å threshold to the bioactive ones. With regard to the apparent potency of bioactive conformations reproduction (35.2% of the minimum RMSD of generated conformations are within 0.5 Å intervals from the bioactive ones) as well as speedup advantage, Cyndi outperforms other methods both in accuracy and efficiency. Table A1 (see Additional file 2) lists more detailed comparisons of Cyndi and other methods for each molecule. The result of initial single 3D conformers generated by ChemAxon's Standardizer module is also tabulated in Table A1 (see Additional file 2) as reference.

**Figure 8:**
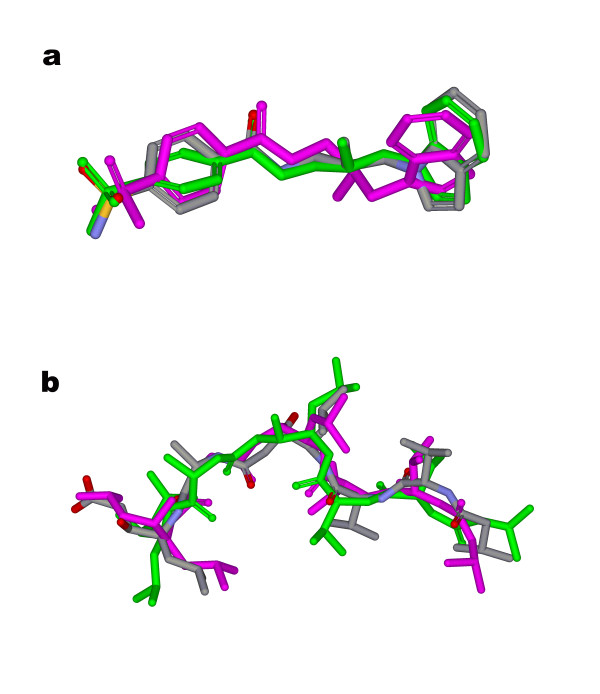
**Comparing the performance of Cyndi with 3 objectives and 4 objectives**. Exemplary alignments of conformers generated by Cyndi with 3 and 4 objectives: (a) 1IF8 and (b) 1SME. Only the best-fitted conformers to the bioactive structures are shown. The crystal structure conformations are coloured in terms of element (Carbon in grey; oxygen in red and nitrogen in blue), the conformers generated by Cyndi with 3 objectives are coloured in magenta and the ones with 4 objectives are in green.

Comparing with 3-objective case, additional objective of gyration radius slightly bettered the result (lowering the average minimum RMSD from 0687 Å to 0.607 Å) and the number of minimum RMSD within 0–0.5 Å cutoff to the bioactive conformers increased from 107 to 116. Essentially, considering the definition of gyration radius and essence of VDW energy, there would be certain overlapping between these two objectives on penalizing the steric bumped conformations, which has been observed (data not shown). This is part of the reason that gyration radius was formulated as an optional objective. Including gyration radius may improve the qualities of generated conformations for some flexible molecules, but it is not always the case. Actually the *Pareto *solutions in 4-objective space do not necessarily bear larger gyration radius because MOEA algorithm never optimizes towards the extremum of any single objective. Figure 8 depicts the alignments of best-fit conformers for two flexible molecules (PDB entries 1if8 and 1sme) generated with and without gyration radius objective. For 1if8, the bioactive structure adopts a relatively extended conformer with a gyration radius of 5.250 Å; comparing with the conformer generated in 3-objective case, the minimum RMSD (to the bioactive one) of conformer generated by Cyndi with 4-objective is reduced from 1.186 Å to 0.501 Å with gyration radius increasing from 4.830 Å to 5.233 Å (see Figure 8a); while for 1sme, the minimum RMSD increases from 1.754 Å to 2.569 Å with gyration radius increasing from 6.093 Å to 6.274 Å for 3-objective and 4-objective cases respectively (see Figure 8b). In fact, additional objective separates the solutions across more refined hyper grids on the *Pareto *frontiers so as to sample the conformational space more thoroughly. For linear molecules, including gyration radius may boost the procedure of conformational sampling if the bioactive conformers adopt more extended conformation; however, maximizing gyration radius in Cyndi may blur the unbiased sampling procedure for highly branched molecules (as 1sme in Figure 8b) which may harbor multiple reasonable spatial extensions. Practically, because of the inherit irreproducibility flaw of evolution algorithm, one is advised to repeat several independent runs from diverse multiple random conformations both with and without gyration radius objective to sample the conformational space more thoroughly, as Table A2 (see Additional file 3) shows, the results from 3 independent runs of Cyndi outperformed that of single run to some extent.

### Assessment geometrical diversity of generated conformer ensembles

**Figure 9:**
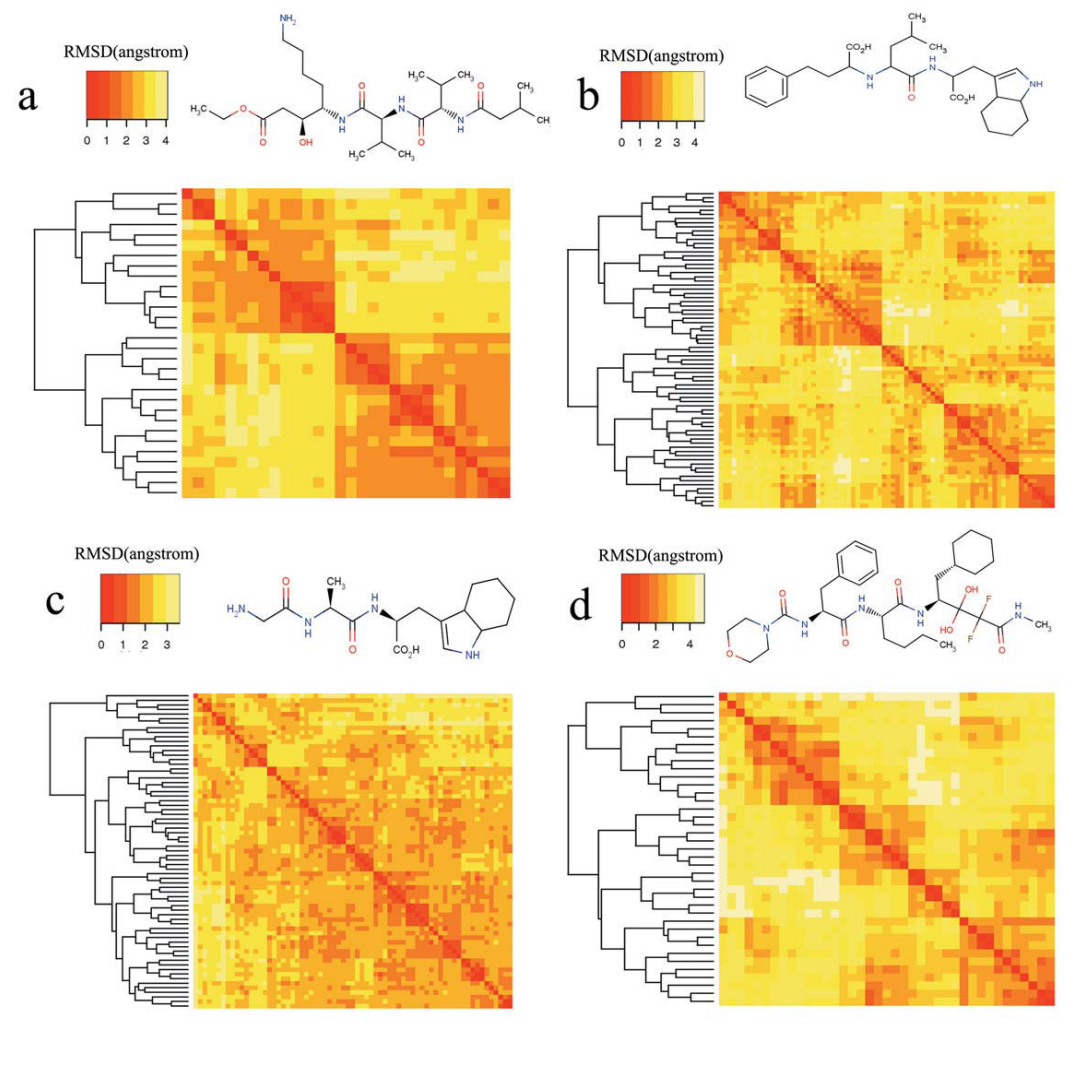
**Clustering analyses of generated conformers for 4 selected molecule structures**. The dendrograms and reordered similarity heatmaps represent the results of the complete-linkage clustering, applying to the distance matrix populated via computing the global RMSD values between each pair of conformers in the ensembles. The blocks in the heatmaps are coloured according to the legend which represents RMSD value as colour bins. The structures for a) 1APT, b) 1TMN, c) 8GCH, and d) 1EPO are presented.

The RMSD filter was applied to remove the conformational redundancy. To quantify the geometrical variation, agglomerative hierarchical clustering analysis was performed on the populated distance matrix via calculating the RMSD values between each pair of conformers. The resulted clustering dendrograms as well as corresponding reordered similarity heatmaps for four selected conformer ensembles are shown in Figure 9. In principle, if the conformers in the ensemble are totally geometrically different from each other, explicit non-overlapping diagonal blocks should emerge from the similarity matrix. The ligand of 1apt (isovaleryl (iva)-val-val-lysta-o-et) is typically a peptide mimic with 3 peptide bonds and a total of 18 rotatable bonds. Since the stiffness induced by the peptide bonds (energy-constrained close to 0 and 180 degrees), the conformer ensemble is generally clustered into two major groups indicated by two large on-diagonal blocks, the members in each cluster are at least within 3.0 Å RMSD threshold against each other (see Figure 9a). Each of the large clusters is divided into much smaller independent ones if we lower the dissimilarity threshold to 0.5 or 1.0 Å, indicating the generated conformers are geometrically dissimilar to each other at this level. Similar results have also been observed in the case of all other three molecules because they all bear one or more peptide bonds. Comparing with 1apt, more distinguishable small-size on-diagonal blocks are exemplified by ligand of 1tmn (N-carboxymethyl dipeptide, 14 rotatable bonds) and that of 1epo (mor-phe-nle-chf-nme, 20 rotatable bonds) in Figures 9b and 9d. The conformers are geometrically distinguishable with each other below the RMSD threshold of 2.0 Å, illustrating the generated conformers are highly distantly spread in conformational space. As for a less flexible case, the ligand of 8gch (gly-ala-trp tripeptide with 9 rotatable bonds) in Figure 9c exhibits a more blurred profile of clustering heatmap below the RMSD threshold of 2.0 Å. Allowing for the more compact conformational space formed by relatively small number of rotatable bonds, the RMSD threshold for dissimilarity should be lowered by reducing the scaling factor in GD filter.

Theoretically, the generated conformers should maximize diversity by distinguishing geometrically with each other completely. Practically, this goal can not be achieved because for a molecule bearing middle level flexibility (like 1epo), the energy-favoured accessible areas are limited with respect to broadly sampled conformational space. Herein many conformers have to be discarded which failed to pass the low-energy filter despite their geometrical dissimilarity. The RMSD threshold in the RMSD filter increases with molecular flexibility, and for those molecules with moderate flexibility (with number of rotatable bonds up to 15), the threshold is about 0.5 Å with the scaling parameter set to 0.1. From the observation of the clustering heatmaps, most of the conformers are geometrically different from each other at the threshold level of 0.5 Å considering the corresponding on-diagonal blocks are small-size and non-overlapped (filled by deepest red colour). When increasing the threshold, the areas of on-diagonal blocks become larger and the corresponding borders turn out fuzzier. Cyndi employs a scalable RMSD threshold instead and achieves a good compromise to generate limited number (usually no more than 100 averagely) of diverse conformers.

It may be argued that the generated conformers can be biased geometrically to mimic the starting conformation because GD objective reflects essentially minimized RMSD between the individuals and initial conformation. Actually, different from single objective GA in which the population evolves towards the extremum in the single objective space exclusively, the optimization essential of Cyndi is to generate a non-dominant solution set in the hyperspace of multiple objectives because no single or a few objectives can exclusively harness the optima towards the extrema in single or parts of objective space. For the *Pareto optimal *conformers in the final archive, most of them are geometrically distant from the initial conformers because they are favourable in other objective spaces such as VDW and torsion energy. Namely the objectives divide the objective hyperspace into grids with the size of corresponding *ο *values and the *Pareto *solutions scatters across those non-dominant grids consisting of *Pareto *frontier.

Currently, Cyndi only supports generating conformer ensemble from an initial 3D conformation, and moreover, since Cyndi merely perturbs the dihedral torsions in optimization procedure, it is not compulsive but highly recommended starting from the conformer converted by the 2D-to-3D conversion toolkits or the one energy-minimized to local minimum with appropriate force field to ensure the generated conformers bear reasonable bond lengths and bond angles. The work of integrating the 1D-to-3D and 2D-to-3D features to perfect Cyndi in handling the problem of conformation generation is under progress.

## Conclusion

A new conformation generation method Cyndi was designed based on a fast and robust MOEA theory. Using multiple objectives controlling energy accessibility as well as geometric diversity, Cyndi is capable of searching the conformational space in nearly constant time and sampling the *Pareto *frontier on which both energy and diversity features are favoured. Three independent objectives (VDW energy, torsion energy and geometrical dissimilarity) are employed and tested against 329 ligands with available crystal determined conformations, the resulted minimum RMSD to bioactive conformers amounts to 0.864 Å in average. The objectives can be implemented easily so it's very simple to customize Cyndi to generate different conformation ensembles which are *pareto optimals *scattering in the hyperspace formed by different objectives.

## Methods

### Overview of Multi-objective Evolution Algorithm

In real life, most optimization problems involve multiple objectives (possibly in conflict), which should be satisfied simultaneously. Generally, optimization with single objective often results in "optimal" solutions against only one objective but unacceptable with respect to the other ones. To solve this trade-off problem, multiple instead of single reasonable solutions should be reserved, incorporating multiple objectives optimized simultaneously. A solution is said to be *Pareto optimal *if it is not dominated by any other solutions in the solution space, which cannot be improved with respect to any objective without worsening at least one of other objectives. The set of all feasible non-dominated solutions is referred to as the non-dominated or *Pareto optimal *solutions and the *Pareto *frontier can be mapped out by ranking the fitness of the *Pareto optimal *solutions in the search space, see references [43-45] for more details.

Through the operation on the population of individuals with inherent parallel mechanism, GA or EA is suitable to solve multi-objective optimization problems. The crossover operator may exploit structures of good solutions with respect to different objectives to create new non-dominated solutions in unexplored parts of *Pareto *front. There are many MOGA and MOEA methods tailored for different practical problems, and generally speaking they are merely different with respect to fitness assignment procedure, elitism, or diversification approaches [46].

We adopt *ο*-MOEA method, a robust algorithm based on the concept of *ο*-domination developed by Deb *et al*. [47,48] as our conformational searching engine. In *ο*-MOEA, two populations co-evolve, one is the general evolving population ***P(t) ***identical to those used in single-objective GA, the other is the "archive" population ***A(t) ***serving as the collection of *Pareto *solutions generated in the *t*th generation. ***A(0) ***is the *ο*-non-dominated solution set of ***P(0) ***after initialization. The crossover operator is applied to two individuals randomly selected from ***P(t) ***and ***A(t)***, respectively, and each new child is compared with the individuals in the two populations by the *ο*-domination method to check if it would be accepted or not. Each individual in the archive is defined as *identification array ***B **as following:

(1)

where  is the minimum probable value of the *j*th objective function and *ο*_*j *_is the user-definable tolerance between two objective values. Identification array divides the whole *j*th objective function space into the hyper-boxes with the size of *ο*_*j*_. If the identification array of the new child dominates any archive individual, then the dominated individual is substituted by the new child; otherwise the new child is discarded. If none of the two conditions is satisfied, the new child is a *ο*-non-dominated solution. Following criteria are used to handle the new child:

1. When the new child and one archive individual share the same identification array **B**, namely they are in the same box, the new child would only be accepted either if it dominates the archive individual or it is nearer to **B**.

2. When the new child doesn't share one identification array **B **with any of archive individuals, the new child would be accepted.

In this way, the distribution of *Pareto optimal *solution set is attained under the condition that each box of the *Pareto *frontier can only be occupied by one solution. The number of *Pareto *optimal solutions can be restrained by scaling the *ο *value.

Similarly, the selection procedure in the population ***P(t) ***is also determined by epsilon domination. If the new child dominates one or more individuals in ***P(t)***, the new child would substitute one of such individuals randomly, or else it should be discarded. If they are non-dominated by each other, one of them would be substituted by the other randomly to fix the size of the population.

### Details of MOEA in Cyndi

#### 2.1 Genome Encoding

Similar to the encoding scheme used in traditional single objective GA operated on molecules, namely the molecules are divided into a set of rigid fragments linked by several rotatable single bonds, Cyndi encodes the genome into a vector of real numbers within the interval of [0, 2π), which represents the torsion angle each rotatable bond to be rotated during the conformation generation. The phenotypes, namely the conformers, are easily translated through rotating the single bond by the values coded in each gene. There have been some reports declaring that reducing the continuous torsion space into discrete one (using torsion grid or biasing the torsion value towards the favourable ranges discovered through mining torsion space) may boost convergence [15], however, this assumption may cause missing some conformational significant areas due to the limited accessible information, for this reason, we still manipulate the genomes in continuous torsion space.

#### 2.2 Optimization Objective Functions

Two terms of potential energy calculated with Tripos force field, VDW and torsion energy, are taken as parts of the objectives for separating the solutions in energy space, during which both bond lengths and bond angles are frozen. The torsion term is directly subjected to the geometric variations during the evolution, consequently it is selected as one of the objectives; the second objective is VDW energy in that it increases exponentially with intensive steric bumps so as to penalize the "ugly" conformers. The format of VDW term is a conventional Lenard-Jones 12-6 function defined in Tripos force field and no cutoff is employed regarding the small size of most drug-size molecules. Since all the energy calculations are performed without considering solvent effects, electrostatic term is also discarded to avoid abnormal compact conformations induced by intra-molecular interaction such as internal hydrogen bonds and salt bridges.

The profile of energy landscape is often described as a zigzagged hypersurface. Two geometrically similar conformers may locate quite distant in energy hypersurface but converge to one minimum after energy optimization. Therefore a mechanism is necessary to promote geometric diversity in the population of evolving conformers; the geometric dissimilarity (GD) between each individual and the input conformation is set as the third objective, to control the geometrical distribution of the *Pareto optimal *solutions. The GD calculation is formulated as eq. (2):

(2)

where *x*_*indiv*, *i *_and *x*_*input*, *i *_are the positions of the *i*th heavy atom in each individual and the input conformer. Here only the positions of the heavy atoms are considered in GD calculation.

Previous study indicated most of the bioactive conformers tend to adopt relatively extended geometries [16], in light of this important observation, Cyndi provides an optional objective to direct the evolution towards generating conformers with different geometrical extension degree. Similar to the work by Izrailev *et al*., the objective that quantifies the degree of extension is formulated by calculating the gyration radius for each molecule, as defined in eq.(3):

(3)

where **x**_*i *_and *m*_*i *_are the position and mass, respectively, of the *i*th atom;  is the geometrical centre of the molecule. This objective may improve the conformational diversity if we have no idea about whether the bioactive conformer is compact or extended.

The multi-objective optimization model of Cyndi consists of a set of *n *parameters (design variables), a set of *l *objective functions, and a set of *m *constraints. Objective functions and constraints are functions of the design variables. It can be formulated mathematically as follows:

(4)

where ***x ***is the design vector ***x ***= {*T*_*b*1_, ⋯, *T*_*bn*_}^T^, in which *T*_*b*1_, ⋯, *T*_*bn *_are the torsion angles of the *n*th rotatable bonds. Accordingly, the constraints for the design variables (***g****(****x****)s*) can be represented as

(5) 0 ≤ *T*_*b*1_, ⋯, *T*_*bn *_< 2*π*

***y ***is the objective vector, which consists of VDW term ***f***_1_(***x***), torsion score ***f***_2_(***x***), and the diversity of geometry ***f***_3_(***x***) or ***f***_4_(***x***), respectively. Note that the maximal problem for GD or gyration radius of each conformation is converted to minimal problem through reciprocal to ensure the most conformational distribution in geometric space. The maximal ***X ***is denoted as the decision space, and ***Y ***is denoted as the objective space.

The objective functions of Cyndi are easy to extend and customize without modifying kernel of the algorithm. By integrating other objectives as constraints, such as the interaction energy of ligand with the binding receptor or pharmacophore model matching, Cyndi would evolve to facilitate structure or ligand based virtual screening easily.

#### 2.3 Archive Post Processing

The final archive contains the optimal solutions on *Pareto *frontier which have been separated by the objectives and are non-dominant to each other. However, the conformers in the archive may be energy-unfavourable or geometrically crowded because they may be suboptimal in the single objective case. Therefore, the final set of conformations is the pruned result of archive members by user-definable energy and RMSD filters.

The conformational energy and geometry are optimized using the conjugated gradient (CG) method with Tripos force field, and all energy terms excluding electrostatic term and torsion term lest inner electrostatic interaction and torsion angle perturbation undermine generated conformation. After energy minimization, following energy cutoff is applied to discard those conformers beyond the energy window:

(6) *E*_*cutoff *_= *E*_*w *_+ *k *× *N*_*rot *_

where *E*_*w *_is a user-definable energy cutoff (default is 20 kcal/mol), *N*_*rot *_is the number of rotatable bonds of current molecule and *k *is the scaling parameter (user-definable and the default value is 0.5). Similar to Balloon's approach, such energy cutoff takes into account of the effect imposed by molecular flexibility and reflects the observed dependence between the local minima and number of rotatable bonds of bioactive conformation [49]. Only those conformers within the energy threshold are labelled as energy-favoured and survived to subsequent RMSD filter.

The conformers survived from the energy filter are submitted to a RMSD filter to remove geometrical duplicates. We adopted the filter scheme defined in Catalyst [50]. Conformers are compared with each other in terms of RMSD and any conformer within the RMSD tolerance (vide infra) of another conformer is discarded:

(7)

where *c *is a user-definable scaling parameter, and *N*_*rot*_, again, is the number of rotatable bonds. The involvement of molecular flexibility can efficiently reduce the geometrical redundancy for highly flexible molecules.

### Testing of Cyndi

The ability for conformation generation of Cyndi was tested against a data set containing 329 drug-sized molecules whose bioactive conformations were extracted from the PDB database (see Additional file 1). This is a combined set of part of CCDC/Astex subset [51,52] used in Balloon's validation and the subset used by Izrailev et.al. [53,54]. To avoid the influences on the generation of conformers from existing crystal structures, single 3D conformer was regenerated for each molecule from 2D structures using the Standardizer module of ChemAxon package [55].

Cyndi test run was performed on each of the molecules in the test set with 200 populations and 200 generations. The probabilities for crossover and mutation operation were set to 0.85 and 0.1, respectively. The relatively larger mutation probability was designated to sample the conformational space more broadly by mutating the existing conformers more frequently. The epsilon values for the four objectives (VDW energy, torsion energy, GD value, and gyration radius) were set as 20 kcal/mol, 5 kcal/mol, 0.2 Å and 0.1 Å, respectively. The maximum iteration for post processing CG minimization was set to 100, and the convergence criterion based on gradient RMS was set to 0.1 kcal·mol^-1^·Å^-1^. No initial optimization against the input conformers was applied and the input conformers were discarded from the final conformer ensembles. Other parameters were set to the default values mentioned previously.

To make a comparison, the Pentium4 processor optimized version of Balloon (version 0.6.6.4641) [56] as well as three conformer generation methods (Catalyst Best, Catalyst Fast and CEASAR) in DS2.0 [57-60] were run against the same test. All default parameters were applied and the maximum number of generated conformers was limited to 100. The initial single 3D conformers generated by Standardizer of ChemAxon were also retained for further comparison (see Table A1 in Additional file 2).

To make an impartial comparison, the minimum RMSD values between the crystal bound conformers and the conformers generated by all methods were calculated with a third-party program, Vrms 1.0, which is an RMSD calculator removing artificial differences caused by symmetry [61]. In this study, a conformation is considered to be successfully reproduced if the RMSD value is less than 0.5 Å, as compared to the original X-ray structure. Balloon, Cyndi, Vrms and DS 2.0 were all run on a workstation with an Intel Pentium Dual Core Processor (2.66 GHz).

Hierarchical clustering was applied to analyze the conformational diversity of each conformer ensemble generated by Cyndi. Vrms calculated RMSD values between each pairs of conformers were used to populate a distance matrix, which was then analyzed by applying complete linkage agglomerative hierarchical clustering. The clustering results were visualized by dendrograms and reordered heatmaps of the distance matrix.

## Authors' contributions

XL designed and validated Cyndi method and also contributed to analysis and data interpretation. FB contributed to the design of method. SO contributed to method validation. HL conceived the idea of the MOEA in conformation generation and co-drafted the manuscript with XL. HL, XW and HJ participated in Cyndi's design, provided direction for its development and revised the subsequent drafts of this manuscript. All authors read and approved the final manuscript.

## Supplementary Material

Additional File 1 **The pre-compiled version of Cyndi (Windows, 32-bit ×86), parameter input file, Tripos force field parameter file and the test set of 329 high-resolution and drug-concerned small molecules determined from X-ray structures in mol2 format.** Click here for file

Additional File 2 **Detailed calculation results for best-fit conformers of 329 crystal structures by Cyndi and other five conformation generation programs.** Click here for file

Additional File 3 **Detail comparison generated conformers from 3 independent runs against 84 structures.** Distribution of the conformers in 3-objective space from 3 independent runs of 1sme viewing along x, y, z axis respectively. Click here for file
