# Supplementary material for: Cyndi: a multi-objective evolution algorithm based method for bioactive molecular conformational generation
Source: BMC Bioinformatics. 2009 Mar 31;10:101. doi: 10.1186/1471-2105-10-101 (PMC2678094; doi:10.1186/1471-2105-10-101)
Supplement: Additional File 2 — Detailed calculation results for best-fit conformers of 329 crystal structures by Cyndi and other five conformation generation programs. [file 1471-2105-10-101-S2.doc]

**Table A3** **- Detailed calculation results for best-fit conformers of 329 crystal structures by Cyndi and other five conformational generation programs.**

| PDB id | Number rotatable bonds | Minimum RMSD to crystal conformation (Å) | | | | | | | | |
| --- | --- | --- | --- | --- | --- | --- | --- | --- | --- | --- |
|  |  | Cyndi 3-obj^a^ | Cyndi 3-obj^b^ | Cyndi 4-obj^a^ | Cyndi 4-obj^b^ | Catalyst Fast | Catalyst Best | Catalyst CEASAR | Balloon | Standardize^c^ |
| 1a28 | 1 | 0.116 | 0.125 | 0.139 | 0.104 | 1.255 | 0.623 | 1.107 | 0.116 | 0.146 |
| 1a42 | 7 | 0.415 | 0.557 | 0.537 | 0.732 | 0.855 | 0.639 | 0.657 | 0.589 | 0.307 |
| 1a4g | 8 | 0.672 | 0.545 | 0.705 | 0.848 | 1.06 | 0.914 | 1.24 | 1.418 | 1.987 |
| 1a4q | 10 | 1.031 | 0.977 | 1.048 | 1.021 | 1.115 | 1.15 | 1.573 | 1.024 | 0.174 |
| 1a6w | 5 | 0.764 | 0.525 | 0.187 | 0.533 | 0.247 | 0.306 | 0.304 | 0.608 | 1.348 |
| 1a9u | 4 | 0.367 | 0.362 | 0.338 | 0.39 | 0.492 | 0.61 | 0.371 | 0.515 | 2.044 |
| 1aaq | 21 | 2.019 | 2.107 | 2.087 | 1.366 | 2.196 | 2.032 | 1.727 | 1.508 | 2.988 |
| 1acl | 11 | 1.293 | 0.979 | 1.002 | 0.847 | 1.363 | 1.275 | 1.225 | 0.771 | 1.939 |
| 1acm | 7 | 0.53 | 0.82 | 0.371 | 0.809 | 1.187 | 1.065 | 1.22 | 0.862 | 1.62 |
| 1aco | 4 | 0.765 | 0.611 | 0.727 | 0.554 | 0.482 | 0.792 | 0.919 | 0.645 | 1.025 |
| 1aec | 15 | 1.193 | 0.858 | 1.241 | 0.915 | 1.103 | 1.146 | 1.096 | 1.489 | 2.593 |
| 1ai5 | 3 | 0.228 | 0.325 | 0.282 | 0.367 | 0.281 | 0.327 | 0.3 | 0.454 | 1.195 |
| 1aoe | 3 | 0.076 | 0.411 | 0.102 | 0.18 | 0.063 | 0.117 | 0.281 | 0.458 | 0.005 |
| 1apt | 21 | 2.03 | 0.951 | 1.239 | 1.134 | 1.791 | 1.612 | 1.804 | 1.316 | 2.354 |
| 1apu | 19 | 1.57 | 1.334 | 1.126 | 1.503 | 1.753 | 1.612 | 1.694 | 1.471 | 2.258 |
| 1aqw | 11 | 0.924 | 0.339 | 0.875 | 1.002 | 1.396 | 1.395 | 1.26 | 1.059 | 2.006 |
| 1ase | 4 | 0.894 | 0.859 | 0.905 | 0.869 | 0.997 | 0.967 | 0.634 | 0.498 | 0.955 |
| 1atl | 10 | 0.931 | 0.886 | 1.151 | 1.07 | 0.949 | 1.069 | 0.791 | 1.065 | 2.032 |
| 1azm | 3 | 0.281 | 0.251 | 0.326 | 0.275 | 0.512 | 0.369 | 0.321 | 1.018 | 0.539 |
| 1b2h | 18 | 1.043 | 1.118 | 0.898 | 0.8 | 1.611 | 1.288 | 1.582 | 1.671 | 3.155 |
| 1b58 | 17 | 1.094 | 0.887 | 1.57 | 1.214 | 1.207 | 1.506 | 1.876 | 1.607 | 3.427 |
| 1b59 | 4 | 0.49 | 0.438 | 0.721 | 0.581 | 0.823 | 0.71 | 1.171 | 0.307 | 1.455 |
| 1b6h | 17 | 1.304 | 0.983 | 1.016 | 0.794 | 1.554 | 1.328 | 1.011 | 1.311 | 2.863 |
| 1b7h | 18 | 1.023 | 1.014 | 1.003 | 1.227 | 1.497 | 1.654 | 1.236 | 1.192 | 3.179 |
| 1b9v | 8 | 0.757 | 0.686 | 0.869 | 0.906 | 0.485 | 0.846 | 0.775 | 0.819 | 2.293 |
| 1baf | 7 | 0.595 | 0.519 | 0.461 | 0.343 | 1.212 | 1.179 | 1.117 | 0.946 | 0.074 |
| 1bbp | 11 | 1.233 | 1.345 | 1.572 | 1.459 | 1.244 | 1.52 | 1.39 | 1.728 | 1.493 |
| 1bgo | 12 | 1.259 | 1.484 | 1.044 | 1.088 | 1.341 | 1.758 | 1.466 | 1.298 | 3.734 |
| 1bju | 5 | 0.528 | 0.449 | 0.476 | 0.386 | 0.541 | 0.576 | 0.394 | 0.686 | 1.523 |
| 1bl7 | 3 | 0.282 | 0.33 | 0.295 | 0.4 | 0.521 | 0.51 | 0.359 | 1.174 | 1.134 |
| 1blh | 6 | 0.626 | 0.546 | 0.769 | 0.726 | 1.374 | 0.721 | 0.47 | 0.764 | 1.23 |
| 1bma | 15 | 1.902 | 1.497 | 1.628 | 1.524 | 1.876 | 1.727 | 1.4 | 1.816 | 2.665 |
| 1bmq | 14 | 1.201 | 1.164 | 1.619 | 1.725 | 1.65 | 1.47 | 1.833 | 1.998 | 3.668 |
| 1bto | 3 | 0.825 | 0.67 | 0.774 | 0.683 | 0.504 | 0.122 | 0.501 | 0.15 | 0.907 |
| 1byb | 10 | 1.874 | 1.33 | 1.408 | 1.226 | 2.854 | 1.883 | 1.76 | 2.109 | 2.118 |
| 1byg | 2 | 0.229 | 0.3 | 0.195 | 0.216 | 0.568 | 0.247 | 0.592 | 0.544 | 0.969 |
| 1c12 | 5 | 0.612 | 0.675 | 0.529 | 0.7 | 0.629 | 0.793 | 0.865 | 0.739 | 1.123 |
| 1c5c | 4 | 0.302 | 0.795 | 0.353 | 0.315 | 0.494 | 0.746 | 0.687 | 0.3 | 1.383 |
| 1c5x | 1 | 0.301 | 0.16 | 0.157 | 0.159 | 0.882 | 0.893 | 0.103 | 0.197 | 0.009 |
| 1c83 | 4 | 0.452 | 0.563 | 0.444 | 0.428 | 0.791 | 0.303 | 0.521 | 0.439 | 0.741 |
| 1cbs | 5 | 0.641 | 0.481 | 0.291 | 0.32 | 0.596 | 0.574 | 0.643 | 0.954 | 1.151 |
| 1cbx | 5 | 0.516 | 0.621 | 0.902 | 0.455 | 0.891 | 0.555 | 0.691 | 0.392 | 1.132 |
| 1cdg | 4 | 1.342 | 1.199 | 1.182 | 1.128 | 1.363 | 0.886 | 0.902 | 0.224 | 1.439 |
| 1cil | 3 | 0.501 | 0.361 | 0.305 | 0.359 | 0.324 | 0.637 | 0.344 | 0.407 | 0.030 |
| 1ckp | 2 | 0.134 | 0.231 | 0.144 | 0.28 | 1.208 | 0.445 | 0.151 | 0.428 | 0.669 |
| 1cle | 21 | 1.358 | 1.692 | 1.714 | 1.725 | 2.213 | 2.444 | 2.651 | 2.383 | 1.994 |
| 1com | 4 | 0.631 | 0.655 | 0.57 | 0.609 | 0.937 | 0.558 | 0.77 | 0.533 | 0.714 |
| 1cps | 5 | 0.848 | 0.322 | 0.28 | 0.313 | 0.785 | 0.776 | 1.079 | 0.484 | 1.439 |
| 1cqp | 7 | 1.195 | 1.218 | 1.234 | 1.215 | 1.342 | 1.275 | 1.226 | 1.617 | 1.982 |
| 1cvu | 14 | 1.334 | 1.29 | 1.336 | 1.513 | 1.394 | 1.435 | 1.228 | 1.402 | 2.547 |
| 1cx2 | 4 | 0.809 | 0.792 | 0.71 | 0.744 | 0.724 | 0.537 | 1.119 | 0.582 | 1.096 |
| 1d0l | 13 | 2.133 | 1.617 | 1.618 | 1.413 | 1.329 | 1.474 | 1.58 | 1.654 | 3.618 |
| 1d3h | 5 | 0.867 | 0.846 | 0.839 | 0.837 | 0.731 | 0.666 | 0.581 | 0.49 | 0.85 |
| 1d4p | 5 | 0.591 | 0.483 | 0.673 | 0.556 | 1.04 | 0.715 | 1.241 | 1.274 | 1.598 |
| 1dam | 6 | 0.832 | 0.842 | 0.594 | 0.493 | 0.63 | 0.874 | 0.905 | 0.472 | 1.109 |
| 1dbb | 1 | 0.252 | 0.276 | 0.629 | 0.251 | 1.267 | 0.603 | 1.08 | 0.266 | 0.386 |
| 1dd7 | 9 | 1.799 | 1.532 | 2.123 | 2.269 | 0.363 | 1.363 | 2.231 | 1.862 | 3.087 |
| 1dg5 | 5 | 0.41 | 0.331 | 0.552 | 0.388 | 0.512 | 0.895 | 0.355 | 0.425 | 1.301 |
| 1dhf | 10 | 1.106 | 0.85 | 1.174 | 0.972 | 1.492 | 1.392 | 1.665 | 1.364 | 2.404 |
| 1did | 2 | 0.434 | 0.487 | 0.538 | 0.516 | 0.588 | 0.402 | 0.522 | 0.323 | 0.55 |
| 1dmp | 8 | 2.009 | 2.117 | 1.942 | 1.845 | 2.073 | 2.635 | 2.67 | 1.269 | 3.843 |
| 1dog | 1 | 0.589 | 0.614 | 0.104 | 0.615 | 0.59 | 0.359 | 0.55 | 0.096 | 0.617 |
| 1dr1 | 2 | 0.257 | 0.119 | 0.147 | 0.122 | 0.153 | 0.163 | 0.154 | 0.161 | 1.374 |
| 1dwb | 1 | 0.331 | 0.223 | 0.342 | 0.224 | 0.029 | 0.048 | 0.231 | 0.276 | 0.007 |
| 1dwc | 11 | 1.188 | 1.417 | 1.607 | 1.866 | 1.672 | 1.595 | 2.339 | 2.394 | 2.334 |
| 1dwd | 11 | 1.031 | 1.261 | 1.533 | 1.391 | 1.658 | 1.691 | 1.734 | 1.63 | 0.689 |
| 1dy9 | 19 | 1.698 | 1.15 | 1.83 | 1.228 | 1.708 | 1.704 | 1.517 | 1.92 | 2.445 |
| 1dyr | 5 | 0.41 | 0.501 | 0.552 | 0.319 | 0.508 | 0.865 | 0.534 | 0.591 | 1.261 |
| 1eap | 11 | 0.899 | 0.95 | 0.993 | 0.925 | 1.013 | 0.993 | 1.033 | 0.992 | 1.94 |
| 1ebg | 3 | 0.31 | 0.33 | 0.331 | 0.329 | 0.782 | 0.342 | 0.339 | 0.52 | 0.858 |
| 1ecv | 4 | 0.607 | 0.465 | 0.502 | 0.54 | 0.477 | 0.428 | 0.471 | 0.517 | 0.795 |
| 1eed | 22 | 2.739 | 2.084 | 2.627 | 2.2 | 1.439 | 2.096 | 1.995 | 1.971 | 4.035 |
| 1ei1 | 8 | 1.018 | 1.037 | 1.323 | 1.264 | 1.295 | 1.807 | 1.269 | 1.258 | 2.186 |
| 1ejn | 7 | 0.937 | 0.71 | 0.764 | 0.948 | 0.794 | 1.308 | 1.443 | 0.641 | 1.803 |
| 1eoc | 1 | 0.063 | 0.897 | 0.066 | 0.063 | 0.904 | 0.903 | 0.216 | 0.217 | 0.054 |
| 1epb | 5 | 0.703 | 0.721 | 0.864 | 0.708 | 1.14 | 0.812 | 0.9 | 0.815 | 1.809 |
| 1epo | 20 | 2.693 | 1.508 | 2.615 | 1.754 | 2.398 | 1.841 | 3.214 | 1.225 | 2.739 |
| 1eta | 5 | 0.596 | 0.61 | 0.386 | 0.758 | 0.946 | 0.693 | 0.796 | 0.465 | 0.278 |
| 1etr | 11 | 1.705 | 1.557 | 1.338 | 1.473 | 1.732 | 1.652 | 2.379 | 2.377 | 2.501 |
| 1ets | 11 | 1.555 | 1.276 | 1.417 | 1.157 | 1.727 | 1.84 | 2.193 | 1.879 | 3.485 |
| 1ett | 8 | 0.929 | 0.472 | 0.906 | 1.014 | 1.026 | 0.838 | 2.226 | 1.675 | 2.256 |
| 1f0r | 5 | 1.366 | 0.831 | 1.21 | 0.931 | 1.406 | 1.274 | 1.59 | 1.358 | 0.015 |
| 1f0s | 5 | 1.241 | 1.178 | 1.192 | 1.172 | 1.652 | 1.143 | 0.98 | 0.698 | 2.833 |
| 1f0u | 11 | 1.705 | 1.518 | 1.131 | 1.174 | 1.305 | 1.492 | 1.66 | 0.973 | 2.46 |
| 1f3d | 3 | 0.449 | 0.383 | 0.422 | 0.413 | 0.527 | 0.596 | 0.398 | 0.651 | 0.012 |
| 1fax | 7 | 0.625 | 1.35 | 1.171 | 0.967 | 1.156 | 0.746 | 1.208 | 2.184 | 0.517 |
| 1fcy | 3 | 0.377 | 0.269 | 0.162 | 0.201 | 0.749 | 0.319 | 0.754 | 0.704 | 1.056 |
| 1fcz | 4 | 0.459 | 0.391 | 0.407 | 0.432 | 0.617 | 0.649 | 0.578 | 0.767 | 0.891 |
| 1fen | 4 | 0.514 | 0.461 | 0.335 | 0.323 | 0.762 | 0.467 | 0.401 | 0.503 | 1.187 |
| 1fgi | 4 | 0.589 | 0.446 | 0.273 | 0.518 | 0.79 | 0.615 | 0.698 | 0.588 | 0.856 |
| 1fkg | 11 | 1.001 | 1.301 | 1.259 | 0.938 | 1.084 | 1.741 | 1.686 | 1.742 | 2.79 |
| 1fkh | 11 | 1.557 | 1.243 | 1.657 | 1.286 | 1.271 | 1.699 | 1.587 | 2.763 | 2.077 |
| 1fl3 | 8 | 0.743 | 0.637 | 0.611 | 0.65 | 0.83 | 0.817 | 0.824 | 0.88 | 0.881 |
| 1flr | 2 | 0.257 | 0.263 | 0.292 | 0.272 | 2.105 | 0.391 | 0.571 | 0.197 | 2.136 |
| 1frb | 5 | 1.051 | 0.779 | 0.77 | 0.962 | 1.063 | 0.688 | 1.026 | 0.931 | 2.59 |
| 1frp | 6 | 0.694 | 0.493 | 0.725 | 0.425 | 0.958 | 1.055 | 1.019 | 0.402 | 1.081 |
| 1ftm | 3 | 0.247 | 0.377 | 0.461 | 0.39 | 0.339 | 0.345 | 0.273 | 0.426 | 1.226 |
| 1g9v | 7 | 0.969 | 0.875 | 0.963 | 0.975 | 0.683 | 0.664 | 0.891 | 0.703 | 1.553 |
| 1gkc | 11 | 1.105 | 0.787 | 0.795 | 0.372 | 0.87 | 1.023 | 1.446 | 0.56 | 2.045 |
| 1glp | 12 | 1.066 | 0.828 | 1.141 | 0.921 | 1.539 | 1.59 | 1.29 | 1.332 | 2.242 |
| 1glq | 15 | 1.506 | 1.197 | 1.516 | 1.289 | 1.33 | 1.642 | 1.26 | 1.276 | 2.285 |
| 1gm8 | 5 | 0.506 | 0.444 | 0.519 | 0.482 | 0.72 | 0.985 | 0.654 | 0.643 | 2.255 |
| 1gr2 | 4 | 0.346 | 0.357 | 0.339 | 0.269 | 0.888 | 0.469 | 0.328 | 0.419 | 0.83 |
| 1hak | 7 | 1.539 | 1.347 | 1.405 | 1.078 | 1.072 | 1.169 | 1.198 | 1.26 | 2.186 |
| 1hdc | 6 | 0.937 | 0.978 | 1.034 | 1.065 | 1.96 | 2.133 | 2.061 | 1.544 | 1.514 |
| 1hef | 23 | 2.171 | 2.153 | 2.139 | 1.966 | 2.203 | 2.029 | 2.28 | 2.654 | 2.935 |
| 1hfc | 12 | 0.664 | 1.052 | 1.097 | 0.887 | 1.277 | 0.455 | 0.912 | 0.637 | 2.051 |
| 1hiv | 25 | 3.065 | 2.369 | 2.734 | 2.759 | 2.416 | 2.145 | 2.571 | 2.812 | 3.06 |
| 1hnn | 1 | 0.137 | 0.466 | 0.178 | 0.302 | 0.478 | 0.172 | 0.117 | 0.338 | 1.034 |
| 1hos | 24 | 2.793 | 2.473 | 2.8 | 2.537 | 2.045 | 2.163 | 2.204 | 2.753 | 3.314 |
| 1hp0 | 2 | 0.696 | 0.722 | 0.738 | 0.75 | 0.62 | 0.65 | 0.342 | 0.464 | 1.959 |
| 1hpv | 13 | 1.937 | 1.517 | 1.562 | 1.669 | 1.57 | 1.53 | 1.525 | 1.585 | 3.238 |
| 1hq2 | 1 | 0.153 | 0.14 | 0.245 | 0.148 | 0.109 | 0.09 | 0.098 | 0.187 | 0.382 |
| 1hri | 9 | 0.976 | 0.644 | 1.042 | 1.09 | 1.148 | 0.888 | 1.026 | 0.902 | 1.559 |
| 1hsb | 8 | 0.453 | 0.453 | 0.654 | 0.489 | 0.856 | 0.788 | 0.828 | 0.719 | 1.63 |
| 1hsl | 3 | 0.155 | 0.11 | 0.31 | 0.146 | 0.47 | 0.558 | 0.812 | 0.678 | 0.895 |
| 1htf | 15 | 1.17 | 1.167 | 1.282 | 1.029 | 1.418 | 1.43 | 1.929 | 2.034 | 3.909 |
| 1hvj | 25 | 3.155 | 2.289 | 3.413 | 2.923 | 2.544 | 3.88 | 3.085 | 3.693 | 2.675 |
| 1hvl | 25 | 3.265 | 2.592 | 2.636 | 2.733 | 2.652 | 2.8 | 3.318 | 2.592 | 2.364 |
| 1hvr | 8 | 2.357 | 2.249 | 2.212 | 2.403 | 2.458 | 2.404 | 2.894 | 3.24 | 4.613 |
| 1hvy | 10 | 1.008 | 0.782 | 1.294 | 0.94 | 1.262 | 1.533 | 1.33 | 1.214 | 2.956 |
| 1hwi | 8 | 1.176 | 0.916 | 1.068 | 1.417 | 1.013 | 0.897 | 0.976 | 0.71 | 2.04 |
| 1hyt | 5 | 0.275 | 0.478 | 0.466 | 0.404 | 0.976 | 0.43 | 0.141 | 0.495 | 1.268 |
| 1ia1 | 2 | 0.387 | 0.306 | 0.271 | 0.258 | 1.755 | 0.495 | 0.17 | 0.153 | 0.008 |
| 1ia3 | 3 | 0.215 | 0.314 | 0.255 | 0.397 | NA | NA | NA | 0.371 | 1.982 |
| 1ian | 4 | 0.309 | 0.239 | 0.345 | 0.245 | 0.481 | 0.594 | 0.321 | 0.425 | 1.291 |
| 1ibg | 4 | 0.803 | 0.908 | 0.885 | 0.752 | 1.455 | 2.172 | 2.288 | 1.459 | 1.204 |
| 1icn | 15 | 1.473 | 1.233 | 1.267 | 1.151 | 0.992 | 1.299 | 1.287 | 1.179 | 1.759 |
| 1ida | 18 | 2.574 | 1.467 | 1.79 | 2.07 | 2.061 | 2.404 | 1.796 | 2.26 | 3.751 |
| 1if8 | 7 | 1.186 | 1.083 | 0.501 | 0.769 | 1.006 | 1.125 | 0.734 | 1.016 | 1.666 |
| 1ig3 | 4 | 0.552 | 0.597 | 0.277 | 0.44 | 0.371 | 0.341 | 0.434 | 0.507 | 1.729 |
| 1imb | 2 | 0.195 | 0.167 | 0.231 | 0.257 | 0.822 | 0.538 | 0.861 | 0.721 | 0.829 |
| 1ivb | 4 | 0.58 | 0.265 | 0.582 | 0.58 | 0.884 | 0.932 | 0.392 | 0.493 | 1.001 |
| 1ivq | 18 | 3.332 | 3.234 | 2.826 | 2.51 | 2.11 | 2.06 | 2.184 | 2.302 | 4.68 |
| 1j3j | 2 | 0.688 | 0.705 | 0.728 | 0.689 | 0.786 | 0.622 | 0.57 | 0.276 | 0.635 |
| 1jap | 10 | 1.114 | 0.732 | 0.896 | 0.55 | 1.217 | 0.895 | 1.008 | 0.913 | 2.157 |
| 1jd0 | 3 | 0.189 | 0.312 | 0.277 | 0.373 | 1.046 | 0.935 | 0.929 | 0.965 | 1.376 |
| 1jje | 7 | 0.485 | 0.385 | 0.441 | 0.72 | 0.886 | 1.133 | 1.142 | 0.422 | 0.729 |
| 1jla | 7 | 0.572 | 0.59 | 0.717 | 0.671 | 0.661 | 0.708 | 0.427 | 0.629 | 1.549 |
| 1k3u | 7 | 0.792 | 0.981 | 0.977 | 0.615 | 0.892 | 1.108 | 1.007 | 0.869 | 2.369 |
| 1ke5 | 4 | 0.552 | 0.344 | 0.419 | 0.399 | 0.624 | 0.477 | 0.496 | 0.565 | 0.998 |
| 1kel | 10 | 1.567 | 1.508 | 1.562 | 1.477 | 1.345 | 1.421 | 1.656 | 1.805 | 2.1 |
| 1kzk | 12 | 2.192 | 1.424 | 1.693 | 1.773 | 1.352 | 2.077 | 1.3 | 1.883 | 2.692 |
| 1l2s | 4 | 0.639 | 0.431 | 0.649 | 0.382 | 0.822 | 0.819 | 0.689 | 0.556 | 0.645 |
| 1l7f | 9 | 0.594 | 0.684 | 1.095 | 0.586 | 0.918 | 0.889 | 0.996 | 0.518 | 0.638 |
| 1lah | 4 | 0.262 | 0.122 | 0.527 | 0.489 | 0.457 | 0.188 | 0.582 | 0.394 | 0.426 |
| 1lcp | 3 | 0.103 | 0.094 | 0.168 | 0.093 | 0.799 | 0.127 | 0.118 | 0.156 | 1.322 |
| 1ldm | 1 | 0.218 | 1.176 | 0.219 | 0.218 | 0.217 | 0.222 | 0.15 | 0.135 | 1.148 |
| 1lic | 15 | 1.196 | 1.437 | 1.544 | 1.05 | 1.228 | 1.074 | 1.09 | 1.205 | 3.165 |
| 1lmo | 8 | 0.56 | 0.712 | 0.758 | 0.721 | 1.258 | 1.018 | 0.982 | 0.859 | 2.707 |
| 1lna | 9 | 0.576 | 0.652 | 0.874 | 0.698 | 1.235 | 1.386 | 0.935 | 0.982 | 1.813 |
| 1lpm | 8 | 0.94 | 0.738 | 0.638 | 0.73 | 0.726 | 0.86 | 0.965 | 0.393 | 2.206 |
| 1lpz | 7 | 0.868 | 0.88 | 1.269 | 0.953 | 0.946 | 0.794 | 1.079 | 1.243 | 0.041 |
| 1lrh | 2 | 0.249 | 0.277 | 0.278 | 0.291 | 0.504 | 0.833 | 0.706 | 0.801 | 0.386 |
| 1lst | 5 | 0.16 | 0.16 | 0.216 | 0.129 | 0.726 | 0.458 | 0.381 | 0.53 | 0.937 |
| 1lyb | 27 | 2.099 | 2.259 | 2.354 | 2.625 | 1.853 | 2.5 | 2.282 | 2.443 | 3.006 |
| 1lyl | 5 | 0.618 | 0.103 | 0.463 | 0.1 | 0.118 | 0.433 | 0.404 | 0.571 | 0.775 |
| 1m2z | 2 | 0.306 | 0.306 | 0.283 | 0.322 | 1.372 | 1.045 | 1.46 | 0.291 | 0.343 |
| 1mcq | 7 | 0.884 | 0.939 | 0.93 | 0.844 | 1.038 | 1.094 | 1.207 | 1.357 | 2.137 |
| 1mcr | 7 | 0.513 | 0.439 | 0.518 | 0.353 | 1.162 | 0.918 | 1.17 | 0.853 | 2.055 |
| 1mdr | 2 | 0.521 | 0.328 | 0.403 | 0.178 | 0.999 | 0.995 | 0.99 | 0.466 | 1.274 |
| 1meh | 6 | 1.251 | 0.364 | 1.094 | 0.919 | 0.877 | 0.464 | 0.787 | 0.836 | 2.716 |
| 1mld | 5 | 0.993 | 0.506 | 0.263 | 0.455 | 0.508 | 0.71 | 0.68 | 0.519 | 1.187 |
| 1mmq | 8 | 1.959 | 1.798 | 1.797 | 1.859 | 1.315 | 1.259 | 1.331 | 1.768 | 2.877 |
| 1mmv | 9 | 0.844 | 0.652 | 0.744 | 0.801 | 1.288 | 0.887 | 1.731 | 0.86 | 0.766 |
| 1mrk | 2 | 1.047 | 0.955 | 0.855 | 0.954 | 0.802 | 0.729 | 0.61 | 0.325 | 1.657 |
| 1mts | 7 | 0.838 | 0.999 | 1.019 | 0.926 | 1.26 | 0.988 | 1.14 | 1.146 | 0.913 |
| 1mtv | 8 | 0.632 | 0.858 | 1.045 | 0.937 | 0.961 | 0.812 | 0.921 | 0.91 | 1.071 |
| 1mtw | 7 | 1.313 | 0.982 | 0.953 | 1.243 | NA | NA | NA | 1.245 | 2.451 |
| 1mup | 2 | 0.549 | 0.3 | 0.321 | 0.285 | 0.484 | 0.404 | 0.412 | 0.331 | 0.7 |
| 1mzc | 7 | 1.349 | 0.996 | 1.337 | 0.992 | 1.705 | 0.903 | 1.813 | 1.775 | 0.015 |
| 1n1m | 3 | 0.291 | 0.129 | 0.353 | 0.156 | 0.226 | 0.283 | 0.418 | 0.276 | 0.568 |
| 1n2j | 3 | 0.136 | 0.159 | 0.159 | 0.117 | 0.517 | 0.628 | 0.586 | 0.18 | 1.179 |
| 1n2v | 3 | 0.424 | 0.259 | 0.276 | 0.224 | 0.449 | 0.33 | 0.206 | 0.502 | 0.317 |
| 1n46 | 4 | 0.53 | 0.376 | 0.372 | 0.371 | 0.473 | 0.642 | 0.371 | 0.397 | 1.972 |
| 1nav | 5 | 0.69 | 0.574 | 0.52 | 0.655 | 0.587 | 0.786 | 0.414 | 0.34 | 1.938 |
| 1nco | 8 | 0.742 | 0.953 | 1.386 | 1.048 | NA | 1.921 | 0.617 | 1.274 | 2.86 |
| 1ngp | 3 | 0.199 | 0.191 | 0.408 | 0.18 | 0.147 | 0.109 | 0.557 | 0.552 | 1.074 |
| 1nis | 5 | 0.446 | 0.464 | 0.911 | 0.88 | 1.513 | 1.262 | 1.324 | 0.756 | 1.192 |
| 1of1 | 2 | 0.562 | 0.713 | 0.678 | 0.551 | 1.185 | 1.237 | 1.253 | 0.253 | 0.71 |
| 1of6 | 3 | 0.275 | 0.133 | 0.195 | 0.116 | 0.484 | 0.615 | 0.443 | 0.23 | 0.820 |
| 1okl | 2 | 0.283 | 0.277 | 0.312 | 0.168 | 0.171 | 0.16 | 0.143 | 0.24 | 0.918 |
| 1okm | 7 | 0.331 | 0.292 | 0.558 | 0.521 | 0.697 | 0.65 | 0.317 | 0.536 | 0.011 |
| 1opk | 4 | 0.561 | 0.406 | 0.359 | 0.522 | 0.871 | 0.544 | 0.504 | 0.516 | 1.889 |
| 1oq5 | 4 | 0.529 | 0.656 | 0.678 | 0.425 | 1.015 | 0.448 | 1.205 | 0.312 | 1.519 |
| 1owe | 4 | 0.359 | 0.304 | 0.373 | 0.355 | 0.781 | 0.476 | 0.371 | 0.614 | 0.011 |
| 1oyt | 4 | 0.337 | 0.323 | 0.286 | 0.377 | 0.713 | 0.479 | 0.963 | 0.569 | 2.058 |
| 1p2y | 1 | 0.277 | 0.444 | 0.258 | 0.254 | 0.364 | 0.385 | 0.215 | 0.423 | 0.61 |
| 1p62 | 2 | 0.869 | 0.747 | 0.875 | 0.766 | 0.432 | 0.466 | 0.5 | 0.374 | 1.012 |
| 1pbd | 1 | 0.103 | 0.048 | 0.038 | 0.046 | 0.031 | 0.042 | 0.031 | 0.276 | 0.038 |
| 1pdz | 3 | 0.392 | 0.346 | 0.548 | 0.219 | 0.617 | 0.542 | 0.31 | 0.529 | 0.969 |
| 1pgp | 7 | 1.049 | 0.942 | 0.816 | 1.101 | NA | NA | NA | 0.84 | 1.785 |
| 1phd | 1 | 0.185 | 0.11 | 0.152 | 0.18 | 0.659 | 0.422 | 0.101 | 0.111 | 0.364 |
| 1phg | 3 | 0.451 | 0.339 | 0.387 | 0.202 | 1.142 | 0.55 | 0.325 | 0.255 | 1.126 |
| 1pmn | 7 | 0.533 | 0.537 | 0.61 | 0.59 | 0.699 | 0.852 | 0.7 | 1.469 | 0.018 |
| 1poc | 23 | 1.667 | 1.686 | 1.777 | 1.563 | 1.945 | 1.622 | 2.691 | 2.264 | 3.563 |
| 1ppc | 11 | 1.376 | 1.211 | 1.191 | 1.035 | 1.492 | 1.688 | 1.985 | 1.371 | 0.216 |
| 1pph | 8 | 1.042 | 1.034 | 0.761 | 0.935 | 1.02 | 1.205 | 1.681 | 1.522 | 1.855 |
| 1ppi | 12 | 1.829 | 0.96 | 0.952 | 0.811 | 2.705 | 2.424 | 2.492 | 2.294 | 2.556 |
| 1pso | 27 | 1.837 | 1.883 | 2.577 | 2.487 | 2.019 | 2.151 | 2.708 | 2.181 | 2.957 |
| 1ptv | 5 | 0.408 | 0.325 | 0.381 | 0.506 | 1.158 | 0.583 | 0.835 | 0.285 | 0.613 |
| 1q1g | 3 | 0.291 | 0.349 | 0.431 | 0.2 | 0.774 | 0.808 | 0.749 | 0.556 | 0.556 |
| 1q4g | 3 | 0.161 | 0.274 | 0.277 | 0.178 | 0.395 | 0.487 | 0.207 | 0.358 | 1.596 |
| 1qbr | 14 | 3.338 | 2.72 | 2.811 | 2.776 | 2.378 | 2.903 | 2.553 | 3.108 | 5.624 |
| 1qbu | 11 | 3.395 | 2.009 | 2.547 | 2.738 | 2.407 | 2.367 | 2.152 | 2.18 | 4.238 |
| 1qcf | 2 | 0.933 | 0.786 | 0.79 | 0.781 | 0.38 | 0.977 | 0.187 | 0.213 | 0.952 |
| 1qft | 2 | 0.385 | 0.578 | 0.401 | 0.526 | 0.668 | 0.464 | 0.644 | 0.183 | 0.5 |
| 1qpe | 2 | 0.305 | 0.217 | 0.28 | 0.243 | 0.604 | 0.564 | 0.393 | 0.339 | 0.977 |
| 1qpq | 2 | 0.803 | 0.376 | 0.479 | 0.356 | 0.897 | 0.766 | 0.528 | 0.402 | 0.966 |
| 1r1h | 10 | 0.796 | 0.995 | 0.874 | 1.072 | 1.358 | 1.113 | 1.274 | 1.054 | 2.034 |
| 1r55 | 11 | 1.389 | 1.012 | 1.008 | 0.817 | 1.263 | 0.711 | 1.121 | 0.571 | 1.516 |
| 1r58 | 10 | 1.291 | 1.174 | 1.798 | 1.261 | 1.229 | 1.027 | 1.159 | 0.983 | 2.802 |
| 1r9o | 3 | 0.234 | 0.172 | 0.213 | 0.199 | 0.2 | 0.181 | 0.432 | 0.22 | 1.787 |
| 1rds | 8 | 1.881 | 1.388 | 1.546 | 1.763 | 1.741 | 1.677 | 1.827 | 1.221 | 2.553 |
| 1rne | 24 | 4.857 | 2.085 | 1.862 | 1.884 | 2.163 | 2.088 | 2.401 | 2.438 | 2.97 |
| 1rnt | 4 | 1.288 | 1.262 | 1.503 | 1.295 | 0.709 | 0.76 | 0.775 | 0.368 | 2.191 |
| 1rob | 4 | 0.557 | 0.563 | 0.635 | 0.361 | 0.806 | 1.009 | 0.806 | 0.62 | 1.007 |
| 1rt2 | 7 | 0.597 | 0.708 | 0.663 | 0.649 | 0.677 | 0.784 | 0.608 | 0.516 | 2.413 |
| 1s19 | 5 | 0.406 | 0.305 | 0.471 | 0.465 | 1.299 | 0.985 | 1.016 | 1.094 | 2.332 |
| 1s3v | 6 | 0.677 | 0.901 | 0.799 | 0.823 | 0.74 | 0.845 | 0.884 | 0.613 | 2.143 |
| 1sg0 | 2 | 0.142 | 0.127 | 0.152 | 0.157 | 0.431 | 0.081 | 0.07 | 0.307 | 0.093 |
| 1sj0 | 6 | 0.585 | 0.642 | 0.561 | 0.725 | 0.78 | 0.979 | 1.155 | 0.617 | 0.913 |
| 1slt | 6 | 1.996 | 1.749 | 1.732 | 1.759 | 1.017 | 1.013 | 0.922 | 0.689 | 2.058 |
| 1sme | 27 | 1.754 | 1.968 | 2.569 | 1.957 | 1.87 | 1.949 | 2.17 | 2.545 | 2.89 |
| 1snc | 6 | 0.943 | 0.847 | 0.859 | 0.909 | 1.035 | 1.151 | 0.987 | 0.486 | 1.334 |
| 1sq5 | 7 | 0.819 | 1.055 | 0.841 | 0.708 | 0.969 | 0.827 | 0.748 | 0.529 | 1.605 |
| 1srj | 3 | 0.123 | 0.264 | 0.285 | 0.147 | 0.746 | 0.55 | 0.23 | 0.661 | 0.184 |
| 1stp | 5 | 0.488 | 0.616 | 0.336 | 0.278 | 0.279 | 0.699 | 0.825 | 0.664 | 1.498 |
| 1t40 | 7 | 0.986 | 1.014 | 1.106 | 0.942 | 1.213 | 0.836 | 0.848 | 1.217 | 2.257 |
| 1t46 | 8 | 1.192 | 1.434 | 1.427 | 0.993 | 1.049 | 1.27 | 1.441 | 1.546 | 0.035 |
| 1t9b | 6 | 0.597 | 0.692 | 1.081 | 0.978 | 0.607 | 0.741 | 0.886 | 0.558 | 2.052 |
| 1tdb | 4 | 0.889 | 0.726 | 0.669 | 0.719 | 0.733 | 0.894 | 1.093 | 0.708 | 1.219 |
| 1tlp | 12 | 1.76 | 1.519 | 1.735 | 1.732 | 1.484 | 1.697 | 2.055 | 2.231 | 2.659 |
| 1tmn | 14 | 1.27 | 1.611 | 1.568 | 1.549 | 2.029 | 1.843 | 1.674 | 1.786 | 2.954 |
| 1tng | 1 | 0.547 | 0.088 | 0.089 | 0.554 | 0.328 | 0.312 | 0.338 | 0.06 | 0.011 |
| 1tnh | 1 | 0.026 | 0.026 | 0.028 | 0.025 | 0.09 | 0.093 | 0.03 | 0.262 | 0.007 |
| 1tni | 4 | 0.09 | 0.096 | 0.038 | 0.093 | 0.115 | 0.563 | 0.339 | 0.272 | 0.006 |
| 1tnl | 1 | 0.039 | 0.018 | 0.058 | 0.022 | 0.596 | 0.627 | 0.589 | 0.103 | 0.017 |
| 1tow | 4 | 0.632 | 0.286 | 0.487 | 0.308 | 0.329 | 0.319 | 0.537 | 0.287 | 1.219 |
| 1tpp | 4 | 0.375 | 0.22 | 0.231 | 0.236 | 1.012 | 0.689 | 0.46 | 0.524 | 1.112 |
| 1trk | 8 | 1.151 | 1.366 | 1.481 | 1.154 | 1.087 | 1.254 | 1.329 | 1.161 | 2.191 |
| 1tt1 | 4 | 0.627 | 0.57 | 0.781 | 0.542 | 0.812 | 0.643 | 0.897 | 0.418 | 1.094 |
| 1tyl | 2 | 0.118 | 0.161 | 0.265 | 0.124 | 1.005 | 0.987 | 1.002 | 0.494 | 0.943 |
| 1tz8 | 4 | 0.337 | 0.299 | 0.299 | 0.309 | 0.404 | 0.269 | 0.206 | 0.384 | 0.427 |
| 1u1c | 6 | 0.78 | 0.481 | 0.681 | 0.805 | 1.066 | 0.369 | 0.742 | 0.381 | 1.359 |
| 1ukz | 4 | 0.804 | 0.815 | 0.829 | 0.918 | 0.878 | 1.014 | 0.9 | 0.604 | 0.865 |
| 1uml | 11 | 1.405 | 1.577 | 1.252 | 1.047 | 1.333 | 1.444 | 1.228 | 1.301 | 1.563 |
| 1unl | 8 | 0.76 | 0.949 | 0.977 | 1.008 | 0.878 | 0.769 | 0.989 | 0.601 | 1.451 |
| 1uou | 2 | 0.658 | 0.675 | 0.671 | 0.668 | 0.589 | 0.762 | 0.464 | 0.423 | 0.051 |
| 1uvs | 12 | 1.287 | 1.004 | 1.289 | 1.564 | 1.646 | 1.652 | 3.1 | 1.547 | 0.055 |
| 1uvt | 8 | 0.718 | 0.768 | 0.712 | 0.64 | 0.746 | 0.733 | 1.093 | 0.746 | 0.016 |
| 1v0p | 8 | 0.86 | 0.821 | 0.832 | 0.866 | 0.814 | 0.858 | 0.773 | 0.837 | 0.944 |
| 1v48 | 6 | 0.544 | 0.572 | 0.668 | 0.568 | 0.793 | 0.73 | 0.996 | 0.492 | 1.024 |
| 1v4s | 5 | 1.022 | 0.815 | 0.951 | 1.015 | 1.44 | 0.614 | 0.832 | 0.946 | 2.431 |
| 1vcj | 8 | 0.585 | 0.478 | 0.587 | 0.739 | 1.519 | 1.119 | 1.086 | 0.637 | 0.042 |
| 1w2g | 2 | 0.682 | 0.672 | 0.674 | 0.65 | 0.753 | 0.724 | 0.766 | 0.481 | 0.835 |
| 1wap | 3 | 0.381 | 0.145 | 0.206 | 0.169 | 0.652 | 0.667 | 0.411 | 0.25 | 0.367 |
| 1x8x | 3 | 0.178 | 0.192 | 0.346 | 0.239 | 0.484 | 0.615 | 0.443 | 0.346 | 0.820 |
| 1xid | 2 | 0.301 | 0.308 | 0.31 | 0.286 | 0.579 | 0.56 | 0.487 | 0.528 | 0.278 |
| 1xie | 1 | 0.3 | 0.301 | 0.685 | 0.461 | 0.535 | 0.368 | 0.47 | 0.32 | 0.664 |
| 1xm6 | 5 | 0.542 | 0.433 | 0.453 | 0.58 | 0.242 | 0.513 | 0.252 | 0.54 | 0.7 |
| 1xoq | 8 | 0.785 | 0.848 | 0.801 | 0.873 | 0.566 | 0.291 | 0.658 | 0.747 | 2.24 |
| 1xoz | 1 | 0.259 | 0.253 | 0.262 | 0.269 | 0.35 | 0.356 | 0.341 | 0.437 | 0.324 |
| 1y6b | 9 | 1.191 | 1.07 | 1.105 | 0.991 | 1.07 | 1.408 | 1.252 | 1.074 | 0.346 |
| 1ydr | 2 | 0.15 | 0.211 | 0.322 | 0.152 | 0.866 | 0.54 | 1.448 | 0.431 | 0.626 |
| 1ydt | 8 | 0.709 | 0.822 | 0.605 | 0.705 | 0.887 | 0.976 | 0.83 | 1.159 | 0.510 |
| 1yee | 11 | 1.384 | 1.402 | 1.245 | 1.137 | 1.447 | 1.112 | 1.165 | 1.201 | 1.846 |
| 1ygc | 12 | 1.327 | 1.529 | 1.198 | 1.172 | 1.597 | 1.696 | 1.855 | 1.495 | 0.035 |
| 1yqy | 6 | 0.587 | 0.609 | 0.61 | 0.527 | 0.548 | 0.714 | 0.637 | 0.982 | 2.175 |
| 1yv3 | 1 | 0.305 | 0.304 | 0.255 | 0.304 | 0.595 | 0.279 | 0.181 | 0.706 | 0.858 |
| 1yvf | 7 | 1.14 | 0.877 | 0.77 | 0.857 | 1.407 | 1.004 | 1.384 | 1.372 | 1.766 |
| 1ywr | 6 | 0.841 | 0.589 | 0.908 | 0.649 | 0.495 | 0.882 | 0.96 | 0.84 | 0.322 |
| 1z95 | 8 | 1.059 | 1.421 | 1.534 | 1.677 | 1.574 | 1.154 | 1.44 | 0.472 | 2.893 |
| 25c8 | 9 | 1.623 | 1.199 | 1.004 | 1.149 | 0.945 | 1.222 | 1.2 | 0.835 | 1.683 |
| 2aad | 4 | 1.013 | 0.822 | 0.9 | 1 | 0.806 | 0.873 | 0.667 | 0.633 | 1.986 |
| 2ack | 2 | 1.013 | 1.013 | 1.023 | 1.024 | 1.059 | 0.332 | 1.06 | 0.489 | 1.67 |
| 2ada | 2 | 0.443 | 0.497 | 0.468 | 0.512 | 0.48 | 0.41 | 0.662 | 0.571 | 0.812 |
| 2ak3 | 4 | 0.758 | 0.734 | 0.894 | 0.684 | 0.886 | 1.239 | 0.988 | 0.635 | 0.713 |
| 2bm2 | 7 | 0.992 | 0.549 | 0.621 | 0.892 | 1.14 | 0.704 | 1.018 | 1.431 | 0.129 |
| 2br1 | 7 | 0.73 | 0.582 | 0.638 | 0.483 | 0.719 | 0.659 | 0.723 | 0.621 | 1.51 |
| 2bsm | 6 | 0.367 | 0.256 | 0.435 | 0.356 | 0.917 | 0.495 | 0.414 | 0.999 | 0.561 |
| 2cgr | 9 | 1.25 | 0.542 | 0.731 | 1.099 | 0.875 | 1.128 | 1.17 | 0.884 | 1.679 |
| 2cht | 2 | 0.296 | 0.282 | 0.294 | 0.298 | 0.368 | 0.525 | 0.433 | 0.515 | 0.273 |
| 2cmd | 5 | 1.032 | 0.188 | 0.528 | 0.375 | 0.606 | 0.701 | 0.746 | 0.43 | 1.236 |
| 2ctc | 3 | 0.217 | 0.254 | 0.228 | 0.202 | 0.434 | 0.441 | 0.453 | 0.252 | 1.225 |
| 2dbl | 6 | 0.684 | 0.673 | 0.602 | 0.526 | 1.666 | 2.033 | 1.072 | 0.697 | 1.231 |
| 2fox | 7 | 1.068 | 0.535 | 1.072 | 0.848 | 0.803 | 0.85 | 0.565 | 0.87 | 1.997 |
| 2gbp | 1 | 0.509 | 0.309 | 0.557 | 0.118 | 0.37 | 0.357 | 0.521 | 0.511 | 0.115 |
| 2h4n | 3 | 0.367 | 0.203 | 0.33 | 0.248 | 0.505 | 0.202 | 0.19 | 0.322 | 1.028 |
| 2ifb | 14 | 1.409 | 0.909 | 1.346 | 0.922 | 1.102 | 1.059 | 1.083 | 1.032 | 2.33 |
| 2izg | 5 | 0.564 | 0.358 | 0.232 | 0.375 | 0.222 | 0.697 | 0.818 | 0.706 | 1.052 |
| 2lgs | 4 | 0.728 | 0.264 | 0.431 | 0.26 | 0.519 | 0.544 | 0.872 | 0.561 | 0.787 |
| 2mcp | 4 | 1.155 | 1.125 | 1.201 | 1.11 | 1.495 | 1.158 | 1.104 | 0.589 | 1.639 |
| 2pcp | 2 | 0.154 | 0.21 | 0.201 | 0.185 | 1.064 | 0.121 | 1.102 | 0.384 | 0.466 |
| 2phh | 1 | 0.044 | 0.052 | 0.044 | 0.044 | 0.065 | 0.053 | 0.068 | 0.411 | 0.063 |
| 2pk4 | 5 | 0.167 | 0.271 | 0.105 | 0.1 | 0.386 | 0.383 | 0.455 | 0.486 | 0.899 |
| 2plv | 15 | 0.933 | 0.918 | 0.567 | 1.155 | 1.161 | 1.126 | 1.404 | 1.027 | 2.973 |
| 2qwk | 7 | 0.287 | 0.257 | 0.449 | 0.219 | 0.88 | 0.888 | 0.916 | 0.639 | 0.019 |
| 2r07 | 8 | 0.823 | 0.745 | 0.592 | 0.918 | 0.852 | 1.06 | 1.003 | 1.1 | 1.246 |
| 2sim | 6 | 0.315 | 0.381 | 0.623 | 0.369 | 0.501 | 0.646 | 0.619 | 0.583 | 0.856 |
| 2tmn | 5 | 0.239 | 0.238 | 0.448 | 0.456 | 0.646 | 0.371 | 0.792 | 0.307 | 1.579 |
| 2tsc | 12 | 1.17 | 0.963 | 1.53 | 1.065 | 1.66 | 1.397 | 1.574 | 1.419 | 2.488 |
| 2yhx | 4 | 0.388 | 0.449 | 0.572 | 0.362 | 0.776 | 0.781 | 0.751 | 0.476 | 1.411 |
| 2ypi | 3 | 0.245 | 0.284 | 0.615 | 0.234 | 0.95 | 0.442 | 0.447 | 0.351 | 0.475 |
| 3cla | 7 | 0.534 | 0.426 | 0.495 | 0.685 | 1.131 | 0.904 | 1.412 | 0.552 | 1.545 |
| 3cpa | 6 | 0.461 | 0.511 | 0.379 | 0.283 | 1.117 | 0.851 | 0.859 | 0.576 | 1.661 |
| 3erd | 4 | 0.306 | 0.221 | 0.249 | 0.194 | 0.328 | 0.184 | 0.242 | 0.862 | 0.627 |
| 3ert | 8 | 0.331 | 0.585 | 0.586 | 0.473 | 0.563 | 0.776 | 0.561 | 1.278 | 0.033 |
| 3gpb | 3 | 0.628 | 0.58 | 0.34 | 0.24 | 0.779 | 1.092 | 0.63 | 0.504 | 1.146 |
| 3hvt | 1 | 0.275 | 0.275 | 0.382 | 0.183 | 0.361 | 0.34 | 0.244 | 0.74 | 0.136 |
| 3std | 6 | 1.099 | 1.14 | 1.026 | 0.947 | 0.714 | 0.667 | 1.09 | 0.802 | 1.845 |
| 3tpi | 7 | 0.291 | 0.402 | 0.361 | 0.365 | 0.94 | 0.846 | 1.14 | 0.453 | 1.496 |
| 4aah | 3 | 0.337 | 0.336 | 0.537 | 0.295 | 0.653 | 0.762 | 0.634 | 0.599 | 0.984 |
| 4cox | 5 | 0.583 | 0.559 | 0.718 | 0.754 | 1.575 | 0.261 | 0.648 | 0.594 | 1.201 |
| 4cts | 3 | 0.538 | 0.244 | 0.21 | 0.165 | 0.389 | 0.673 | 0.342 | 0.205 | 1.237 |
| 4dfr | 10 | 1.286 | 0.816 | 0.969 | 1.287 | 1.174 | 1.154 | 1.468 | 1.254 | 2.658 |
| 4est | 15 | 1.645 | 0.947 | 1.261 | 1.264 | 1.223 | 1.752 | 1.817 | 1.863 | 2.377 |
| 4fbp | 4 | 0.36 | 0.776 | 0.756 | 0.365 | 0.583 | 1.2 | 0.819 | 0.613 | 0.982 |
| 4lbd | 5 | 0.458 | 0.454 | 0.704 | 0.72 | 0.935 | 1.045 | 0.446 | 0.772 | 2.25 |
| 4phv | 14 | 2.032 | 2.092 | 2.295 | 1.852 | 2.163 | 2.192 | 2.027 | 1.633 | 2.695 |
| 5abp | 1 | 0.15 | 0.15 | 0.146 | 0.148 | 0.16 | 0.398 | 0.297 | 0.133 | 1.087 |
| 5er1 | 19 | 1.707 | 1.495 | 1.999 | 1.545 | 1.78 | 1.621 | 1.404 | 1.476 | 2.529 |
| 5p2p | 21 | 3.607 | 2.512 | 1.996 | 2.002 | NA | NA | NA | 1.95 | 3.644 |
| 5std | 5 | 1.013 | 0.69 | 0.694 | 0.951 | 0.513 | 0.757 | 0.893 | 0.468 | 1.214 |
| 6rnt | 4 | 0.589 | 0.531 | 0.606 | 0.547 | 0.693 | 0.685 | 0.68 | 0.382 | 1.211 |
| 6std | 5 | 0.16 | 0.468 | 0.237 | 0.531 | 0.803 | 0.553 | 1.154 | 0.198 | 2.421 |
| 7std | 5 | 0.235 | 0.311 | 0.395 | 0.333 | 0.495 | 0.623 | 0.765 | 0.403 | 2.148 |
| 7tim | 4 | 0.686 | 0.431 | 0.3 | 0.273 | 0.393 | 0.671 | 0.44 | 0.388 | 1.201 |
| 8gch | 9 | 0.49 | 0.484 | 0.697 | 0.69 | 1.168 | 0.832 | 1.136 | 0.741 | 2.352 |
| Average | 7 | 0.864 | 0.765 | 0.831 | 0.779 | 0.99 | 0.946 | 0.991 | 0.889 | 1.505 |

^a^ Results from 1 run with Cyndi.

^b^ Results from 3 independent runs with Cyndi.

^c^ Results from single 3D conformations generated by Standardize of ChemAxon using 2D structure as inputs.

NA: Fail to generate conformations.
