# Supplementary material for: Cyndi: a multi-objective evolution algorithm based method for bioactive molecular conformational generation
Source: BMC Bioinformatics. 2009 Mar 31;10:101. doi: 10.1186/1471-2105-10-101 (PMC2678094; doi:10.1186/1471-2105-10-101)

**Table A2 - Detail comparison** for Cyndi generated conformers from 3 independent runs

| PDB ID | Num. Rotatable Bonds | Minimum RMSD to crystal conformer (Å) | | | Size of output conformer ensemble | | | Minimum RMSD to crystal conformer (5 multiple runs) (Å) |
| --- | --- | --- | --- | --- | --- | --- | --- | --- |
|  |  | Run 1 | Run 2 | Run 3 | Run 1 | Run 2 | Run 3 |  |
| 1a28 | 1 | 0.138 | 0.267 | 0.118 | 3 | 3 | 4 | 0.132 |
| 1aaq | 21 | 1.954 | 2.062 | 1.983 | 19 | 50 | 18 | 1.712 |
| 1apt | 21 | 1.13 | 1.68 | 1.962 | 29 | 45 | 15 | 1.577 |
| 1apu | 19 | 1.41 | 1.647 | 1.915 | 27 | 56 | 11 | 1.277 |
| 1atl | 10 | 1.354 | 1.065 | 1.551 | 28 | 53 | 12 | 1.132 |
| 1b1h | 18 | 1.212 | 1.478 | 1.298 | 30 | 68 | 5 | 0.883 |
| 1b2h | 18 | 0.907 | 1.03 | 1.178 | 11 | 75 | 33 | 0.673 |
| 1b6h | 17 | 1.076 | 1.234 | 0.961 | 39 | 77 | 39 | 0.803 |
| 1b7h | 18 | 1.181 | 1.071 | 1.199 | 30 | 69 | 7 | 1.05 |
| 1bju | 4 | 0.585 | 0.383 | 0.527 | 62 | 39 | 60 | 0.291 |
| 1bto | 3 | 0.739 | 0.739 | 0.801 | 15 | 21 | 16 | 0.825 |
| 1c83 | 4 | 0.51 | 0.463 | 0.522 | 39 | 39 | 24 | 0.513 |
| 1cbs | 5 | 0.366 | 0.797 | 0.653 | 73 | 29 | 62 | 0.348 |
| 1cbx | 5 | 0.345 | 0.884 | 0.618 | 30 | 74 | 46 | 0.43 |
| 1d3h | 3 | 1.175 | 1.328 | 1.149 | 58 | 24 | 52 | 1.207 |
| 1dam | 6 | 0.589 | 0.77 | 0.909 | 26 | 68 | 28 | 0.692 |
| 1dyr | 5 | 0.365 | 0.573 | 0.489 | 59 | 77 | 71 | 0.311 |
| 1eap | 11 | 0.865 | 0.946 | 1.057 | 69 | 85 | 62 | 0.786 |
| 1ecv | 4 | 0.552 | 0.374 | 0.54 | 46 | 40 | 38 | 0.506 |
| 1eed | 22 | 2.874 | 2.249 | 2.07 | 16 | 34 | 30 | 2.319 |
| 1ejn | 6 | 0.942 | 1.101 | 0.962 | 83 | 61 | 71 | 0.764 |
| 1epo | 20 | 2.179 | 2.063 | 3.25 | 19 | 31 | 14 | 1.664 |
| 1eta | 5 | 0.515 | 0.57 | 0.665 | 76 | 82 | 54 | 0.378 |
| 1etr | 10 | 1.652 | 1.277 | 0.91 | 44 | 55 | 33 | 0.815 |
| 1ett | 8 | 0.788 | 1.046 | 0.903 | 52 | 77 | 61 | 0.771 |
| 1f0u | 9 | 0.937 | 1.208 | 1.107 | 19 | 83 | 47 | 1.432 |
| 1fcy | 3 | 1.585 | 1.321 | 1.768 | 50 | 44 | 40 | 1.301 |
| 1fcz | 4 | 1.165 | 1.172 | 1.114 | 66 | 43 | 68 | 1.028 |
| 1fkg | 11 | 1.175 | 1.106 | 1.051 | 64 | 84 | 47 | 0.928 |
| 1fkh | 11 | 1.648 | 1.083 | 1.536 | 4 | 76 | 36 | 1.497 |
| 1frb | 5 | 1.105 | 1.022 | 0.936 | 83 | 85 | 83 | 0.822 |
| 1ftm | 3 | 0.27 | 0.774 | 0.538 | 35 | 38 | 27 | 0.236 |
| 1glp | 12 | 1.158 | 1.176 | 0.961 | 37 | 57 | 60 | 0.601 |
| 1gr2 | 4 | 1.143 | 0.638 | 1.099 | 68 | 42 | 56 | 1.067 |
| 1hef | 23 | 2 | 1.646 | 3.5 | 14 | 31 | 14 | 1.453 |
| 1hfc | 12 | 1.268 | 0.858 | 0.4 | 40 | 58 | 25 | 0.66 |
| 1hri | 9 | 0.973 | 1.113 | 1.16 | 55 | 70 | 37 | 0.755 |
| 1htf | 15 | 1.158 | 1.556 | 1.356 | 36 | 50 | 19 | 1.302 |
| 1hvj | 25 | 4 | 2.143 | 2.692 | 1 | 44 | 17 | 2.059 |
| 1hvl | 25 | 2.919 | 3.191 | 3.612 | 15 | 38 | 16 | 2.457 |
| 1hvr | 8 | 1.912 | 1.23 | 1.823 | 73 | 94 | 66 | 1.627 |
| 1ia3 | 3 | 0.226 | 0.296 | 0.227 | 44 | 49 | 42 | 0.306 |
| 1ian | 4 | 0.359 | 0.604 | 0.252 | 52 | 28 | 40 | 0.255 |
| 1icn | 15 | 1.329 | 1.279 | 1.154 | 38 | 97 | 52 | 1.236 |
| 1ida | 18 | 1.975 | 1.762 | 1.912 | 14 | 32 | 22 | 1.696 |
| 1if8 | 7 | 0.209 | 0.269 | 0.448 | 43 | 74 | 58 | 0.557 |
| 1lic | 15 | 1.298 | 1.348 | 1.286 | 19 | 95 | 31 | 1.13 |
| 1lmo | 8 | 0.736 | 0.955 | 2.806 | 24 | 64 | 3 | 0.4 |
| 1lna | 9 | 1.296 | 1.105 | 1.04 | 14 | 69 | 26 | 0.874 |
| 1lst | 5 | 0.61 | 0.644 | 0.501 | 42 | 40 | 28 | 0.283 |
| 1mcr | 7 | 0.867 | 0.502 | 0.498 | 40 | 62 | 34 | 0.468 |
| 1mtv | 7 | 1.161 | 0.806 | 0.961 | 54 | 69 | 71 | 0.692 |
| 1mtw | 7 | 1.533 | 1.109 | 1.554 | 74 | 87 | 66 | 0.666 |
| 1pgp | 7 | 1.264 | 1.314 | 1.331 | 63 | 71 | 46 | 1.027 |
| 1phg | 3 | 0.258 | 0.608 | 0.61 | 50 | 28 | 52 | 0.178 |
| 1poc | 23 | 2.677 | 2.225 | 2.545 | 20 | 42 | 38 | 1.575 |
| 1ppc | 11 | 1.149 | 1.976 | 2.446 | 50 | 67 | 64 | 1.316 |
| 1pph | 8 | 0.991 | 1.862 | 1.566 | 60 | 87 | 59 | 1.24 |
| 1qft | 2 | 0.566 | 0.527 | 0.573 | 5 | 8 | 5 | 0.588 |
| 1rne | 24 | 2.56 | 2.483 | 3.093 | 30 | 44 | 7 | 1.935 |
| 1sme | 27 | 2.454 | 2.485 | 3.422 | 27 | 53 | 7 | 2.159 |
| 1stp | 5 | 0.363 | 0.721 | 0.552 | 58 | 61 | 42 | 0.278 |
| 1tmn | 14 | 1.48 | 1.446 | 1.271 | 56 | 85 | 28 | 0.752 |
| 1tng | 1 | 0.051 | 0.56 | 0.368 | 1 | 2 | 1 | 0.379 |
| 1tnh | 1 | 0.527 | 0.224 | 0.19 | 2 | 3 | 3 | 0.052 |
| 1tni | 4 | 0.368 | 0.509 | 0.103 | 49 | 24 | 36 | 0.38 |
| 2cgr | 7 | 0.967 | 1.052 | 1.181 | 48 | 48 | 56 | 0.888 |
| 2cmd | 5 | 0.471 | 0.443 | 0.494 | 52 | 69 | 61 | 0.433 |
| 2izg | 5 | 0.599 | 0.62 | 0.542 | 33 | 53 | 43 | 0.369 |
| 2lgs | 4 | 0.372 | 0.436 | 0.398 | 37 | 31 | 35 | 0.391 |
| 2plv | 15 | 1.081 | 1.011 | 1.25 | 19 | 91 | 37 | 0.697 |
| 2r07 | 8 | 0.791 | 1.024 | 1.01 | 33 | 90 | 17 | 0.84 |
| 2sim | 6 | 0.527 | 0.344 | 0.493 | 13 | 62 | 16 | 0.368 |
| 2yhx | 4 | 0.818 | 0.84 | 0.809 | 45 | 40 | 47 | 0.706 |
| 3cpa | 6 | 1.754 | 0.747 | 1.175 | 20 | 72 | 32 | 0.917 |
| 3std | 7 | 0.617 | 0.61 | 1.026 | 41 | 76 | 63 | 0.768 |
| 3tpi | 7 | 0.774 | 0.718 | 0.516 | 33 | 80 | 25 | 0.48 |
| 4dfr | 10 | 1.709 | 0.659 | 1.959 | 65 | 68 | 46 | 1.425 |
| 4phv | 14 | 2.615 | 2.489 | 1.981 | 52 | 39 | 15 | 1.756 |
| 5p2p | 21 | 2.962 | 2.158 | 2.615 | 14 | 41 | 44 | 2.107 |
| 5std | 5 | 0.678 | 0.715 | 1.264 | 76 | 78 | 63 | 0.4 |
| 6std | 5 | 0.294 | 0.276 | 0.232 | 68 | 60 | 57 | 0.344 |
| 7std | 5 | 0.222 | 0.382 | 0.467 | 32 | 67 | 21 | 0.313 |
| 8gch | 9 | 0.685 | 0.588 | 0.495 | 61 | 87 | 30 | 0.33 |

**Figure A1**. Distribution of the conformers in 3-objective space from 3 independent runs of 1sme viewing along x, y, z axis respectively.


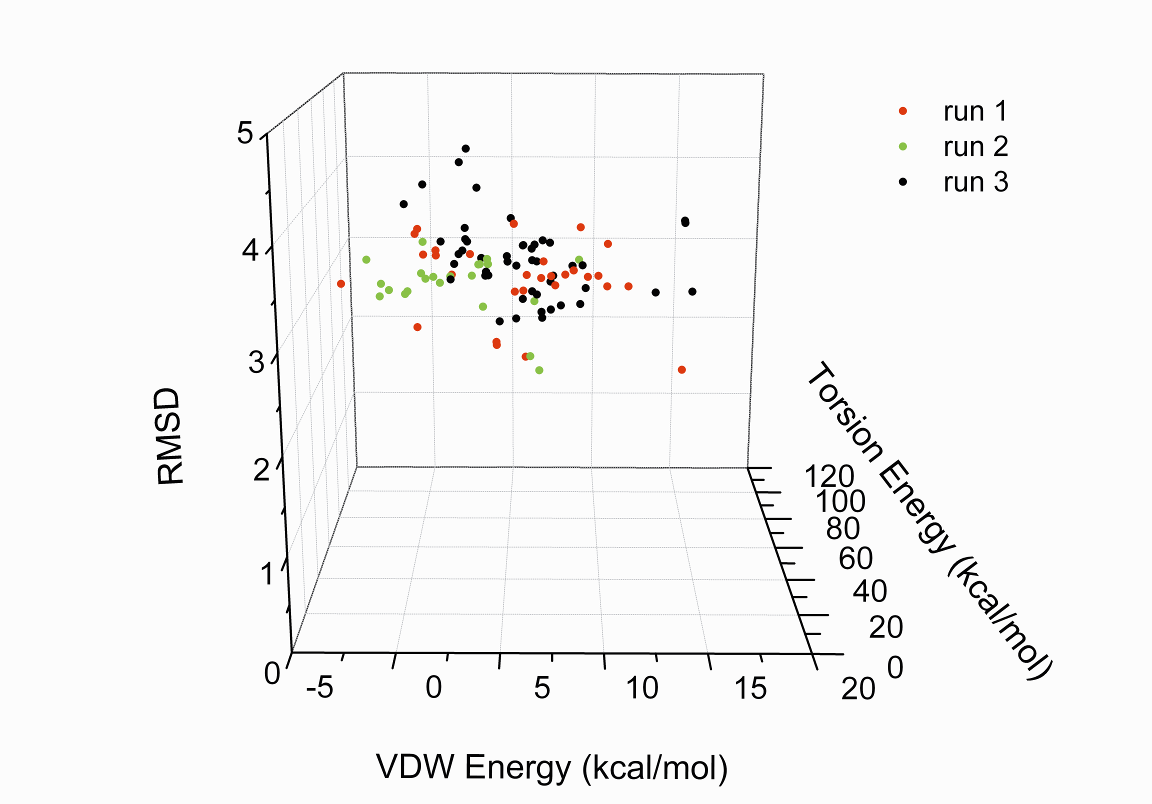

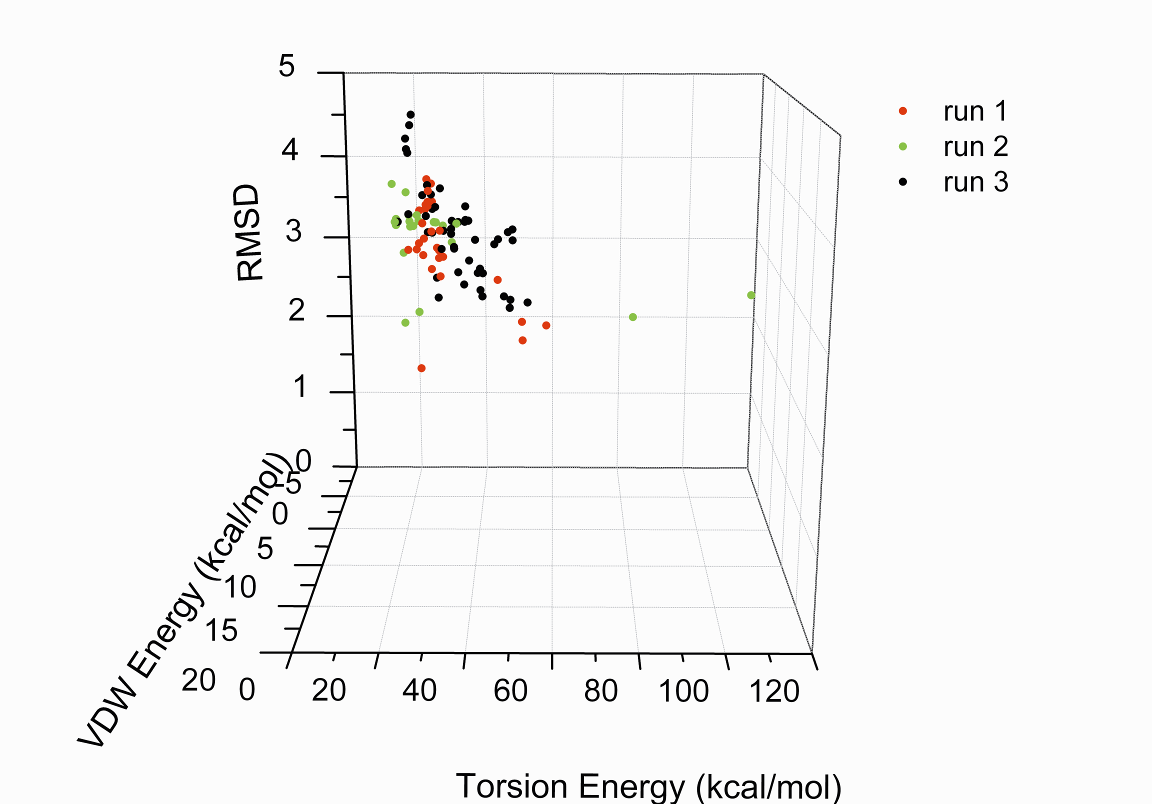

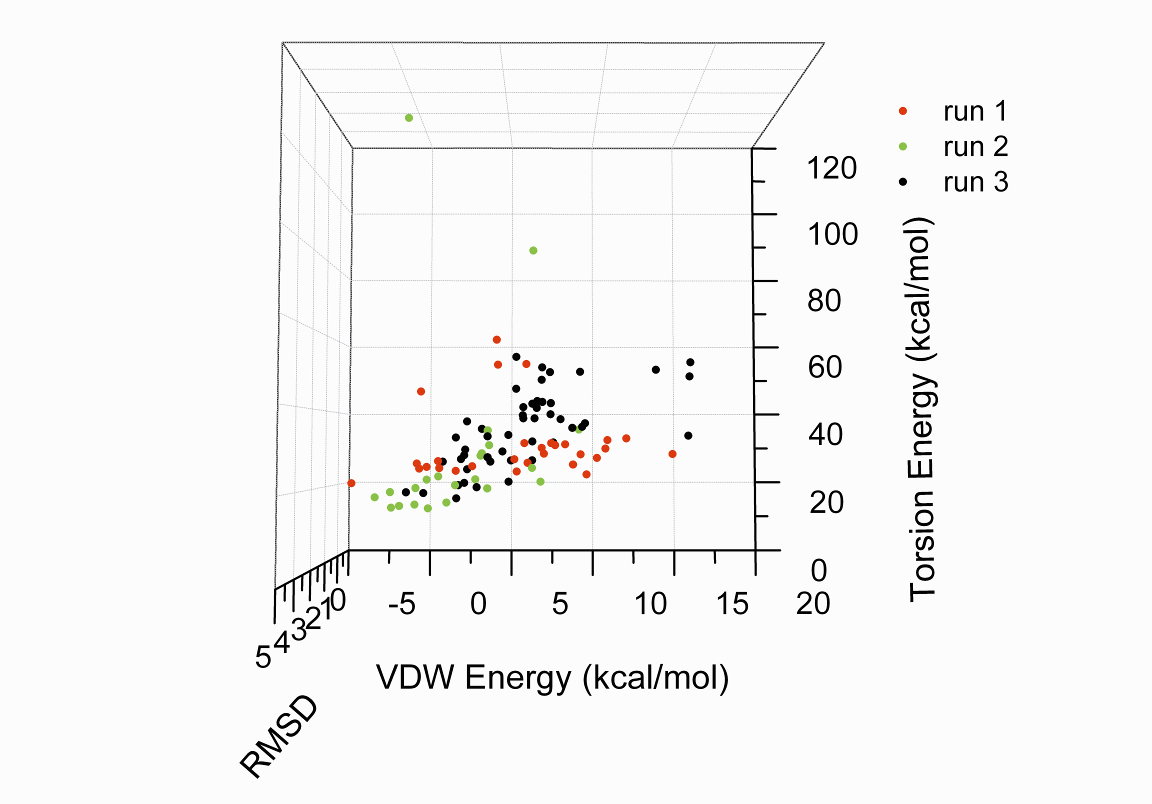

Supplement: Additional File 3 — Detail comparison generated conformers from 3 independent runs against 84 structures. Distribution of the conformers in 3-objective space from 3 independent runs of 1sme viewing along x, y, z axis respectively. [file 1471-2105-10-101-S3.doc]
